# A scoping review on what constitutes a good research culture

**DOI:** 10.12688/f1000research.147599.1

**Published:** 2024-04-23

**Authors:** Amanda Jane Blatch-Jones, Kay Lakin, Sarah Thomas

**Affiliations:** 1 School of Healthcare Enterprise and Innovation, University of Southampton, Southampton, England, SO16 7NS, UK; 2 Hatch, School of Healthcare Enterprise and Innovation, University of Southampton, Southampton, England, SO16 7NS, UK

**Keywords:** research culture, research institutions, funding organisations, academia, open research, early career researchers, transparency, research integrity

## Abstract

**Background:**

The crisis in research culture is well documented, covering issues such as a tendency for quantity over quality, unhealthy competitive environments, and assessment based on publications, journal prestige and funding. In response, research institutions need to assess their own practices to promote and advocate for change in the current research ecosystem. The purpose of the scoping review was to explore ‘ *What does the evidence say about the ‘problem’ with ‘poor’ research culture, what are the benefits of ‘good’ research culture, and what does ‘good’ look like?’*

**Aims:**

To examine the peer-reviewed and grey literature to explore the interplay between research culture, open research, career paths, recognition and rewards, and equality, diversity, and inclusion, as part of a larger programme of activity for a research institution.

**Methods:**

A scoping review was undertaken. Six databases were searched along with grey literature. Eligible literature had relevance to academic research institutions, addressed research culture, and were published between January 2017 to May 2022. Evidence was mapped and themed to specific categories. The search strategy, screening and analysis took place between April-May 2022.

**Results:**

1666 titles and abstracts, and 924 full text articles were assessed for eligibility. Of these, 253 articles met the eligibility criteria for inclusion. A purposive sampling of relevant websites was drawn from to complement the review, resulting in 102 records included in the review. Key areas for consideration were identified across the four themes of job security, wellbeing and equality of opportunity, teamwork and interdisciplinary, and research quality and accountability.

**Conclusions:**

There are opportunities for research institutions to improve their own practice, however institutional solutions cannot act in isolation. Research institutions and research funders need to work together to build a more sustainable and inclusive research culture that is diverse in nature and supports individuals’ well-being, career progression and performance.

**Abbreviations**
AI: Artificial Intelligence
CCB: Complementary Capacity Building
COPE: Committee of Publication Ethics
COS: Center for Open Science
COVID-19: Coronavirus Disease
CRediT: Contributor Roles Taxonomy
CV: Curriculum Vitae
DEI: Diversity Equity and Inclusion
DORA: Declaration on Research Assessment
ECR: Early Career Researchers
EDI: Equality Diversity and Inclusion
ENRI: European Network of Research Integrity
FAIR: Findable, Accessible, Interoperable and Reusable
FTC: Fixed Term Contract
HEI: Higher Education Institution
HEP: Higher Education Provider
INORMS SCOPE: International Network of Research Management Societies (INORMS)
ISRIA: International School on Research Impact Assessment
JBI: Joanna Briggs Institute
JSTOR: Journal Storage
KPI: Key performance Indicator
LMIC: Low Middle-Income Countries
MyNRMN: National Research Mentoring Network
NA: Not applicable
NHS: National Health Service
NIC: Networked Improvement Community
NRMN: National Research Mentoring Network
OA: Open Access
OKI: Open Knowledge Institutions
OIS: Open Innovation Science
ORBIT: ORCID’s Reducing Burden and Improving Transparency
ORCID: Open Researcher and Contributor IDentifier
OSF: Open Science Framework
PEER: Persons Excluded from science because of Ethnicity and Race
PhD: Doctor of Philosophy
PRISMA-ScR: Preferred Reporting Items for Systematic Reviews and Meta-analyses extension for Scoping Reviews
QPP: Quality Publication Practice
REF: Research Excellence Framework
RFO: Research Funding Organisations
RoRi: Research on Research Institute
RPO: Research Performing Organisations
RRP: Responsible Research Practice
STEM: Science, Technology, Engineering, Mathematics
STEMM: Science, Technology, Engineering, Mathematics and Medicine
TOP: Transparency and Openness Promotion
UK: United Kingdom
UKRI: UK Research and Innovation
UKRN: UK Reproducibility Network
USA: United States of America
WoS: Web of Science

## Background

Concerns about the pressures of working in research and the potential negative impact of a poor research culture are well documented in academic literature across diverse disciplines. ^1^^,^^2^ There is a strong connection between concerns about research culture and the inappropriate use of metrics and indicators that drive both institutional and individual researcher behavior, assessment and reward. ^3^^–^^5^ In response to these concerns, a number of actions have emerged to enable and encourage the adoption of a healthier research culture. ^6^^–^^8^ International action to address the underlying drivers of poor research culture include INORMS SCOPE framework for responsible research evaluation ^9^; Declaration on Research Assessment (DORA) ^10^; development of 10 principles for the measurement of research performance: the Leiden Manifesto for Research Metrics ^11^; establishment of the International School on Research Impact Assessment (ISRIA) ^12^; and the HuMetricsHSS Initiative. ^13^

In response to concerns about the experience of working in research, the Wellcome Trust undertook work in the UK to better understand research culture, which has enabled initiatives from the Russell Group and the Royal Society to actively work towards enabling researchers to ‘flourish’. ^14^ A survey conducted by the Wellcome Trust, focused on the experience of researchers, revealed that poor research culture is leading to unhealthy competition, bullying and harassment, mental health issues, and a system that favours quantity over quality. ^15^ Unfortunately, these experiences mirror previous findings, and show the longevity of the issues as the research environment continues to be pressured, competitive and uncertain for many researchers. ^16^

The consequences of poor research culture does not only impact researchers, it also effects research support staff (e.g., technicians, research managers and administrative staff), the production and quality of research, reduces innovation in research and affects public trust in research. ^17^^–^^21^ Funding organisations such as UK Research and Innovation (UKRI) enhanced its 2021-2022 allocation of research culture funding to Higher Education Providers (HEPs) to further explore research processes and experiences of working in research, through piloting new initiatives or enhancing existing activities. ^22^

Striving for excellence and changing research culture is a collective responsibility, requiring action from research institutions, funding organisations and researchers. ^14^ Higher Education Institutions (HEIs) need to assess their own practices to promote and advocate for change in the current research ecosystem. As highlighted by the Wellcome Trust and others, there remains a tendency for quantity over quality, assessment based on publications, journal prestige and funding. ^5^^,^^15^^,^^23^^,^^24^ Any attempts on reform requires commitment from everyone (e.g., publishers, research institutions, funders, researchers etc.) so that diversity, impact, teamwork, open research, and assessment systems are valued. In turn, we may begin to see enhancements for the promotion of transparency, open access, knowledge mobilisation and collaborative networking practices.

The consequences and challenges associated to an inadequate research culture is well evidenced across the research ecosystem and several reports, from funding organisations to independent providers, demonstrating the extent of the problem (for whom and in what context) and the need for a cultural change in research. ^14^^,^^15^^,^^25^^,^^26^ However, the evidence very much focuses on the challenges and barriers, with limited evidence on solutions or how to implement change, initiate opportunities, or what works for whom and in what context that is fit for purpose for all individuals (inclusive of all research and non-research staff in an academic environment). ^27^^–^^29^

The purpose of this scoping review was to explore the evidence on what constitutes a good research culture and how open research, career research paths (recognition and awards) and equality, diversity, and inclusion interplay to enhance and promote a more sustainable research culture environment. The scoping review was intended to inform future practice within a specific research organisation (the University of Southampton, UK), recognising the broader interest in and application of the findings. The scoping review was conducted to address the following question: *What does the evidence say about the ‘problem’ (barriers, challenges, consequences etc.) with ‘poor’ research culture, and what are the benefits of ‘good’ research culture, and what does it look like?*

## Methods

Scoping reviews are relevant to addressing research questions that seek to identify priorities for research, clarification on concepts and definitions, identifying research frameworks, or locating background information in preparation for a systematic review. Scoping reviews aim to understand *‘What has been done previously?’* and *‘What does the literature say?’* compared to systematic reviews that ask the question *‘Does this intervention work for this group of individuals?’* The purpose of this scoping review was to identify the current evidence and body of relevant literature using the Joanna Briggs Institute (JBI) guidance/approach to guide the development, analysis and write up of the scoping review. ^30^^–^^32^ Using this approach enabled the reviewers to map the evidence to four key areas highlighted from existing published work from the Wellcome Trust, to ensure consistency and continuity to predefined areas already established by the research environment. ^15^

### Eligibility criteria

**Context:** The context included UK and international settings within the academic environment (research ecosystem).

**Participants:** Academic, administrative, and technical staff and students of all levels, grades, disciplines, and professions. To be inclusive of academic and non-academic staff to ensure an inclusive approach and incorporating the principles of ‘team science’ and organisational culture.

**Inclusion criteria:** Evidence from research institutions only (considering Education, Enterprise and Research, the triple helix approach ^33^^,^^34^) for both academic and grey literature were included. All disciplines within the academic environment were included.

**Exclusion criteria**: Anyone undertaking or supporting research outside of a research institution/Higher Education Institutes environment (for example in the health and social care field the National Health Service (NHS) Trusts, hospital settings, primary health care settings, allied health professional settings). Industry and non-academic businesses (including consultancies) were not included as they were not considered to have an academic focus. Non-English articles were excluded if no translation was available for the full article.

The database searches and grey literature did not have any limitations on country of origin, apart from news items that were restricted to the UK, Europe, North America, and Australasia.

### Types of sources

The scoping review considered all types of study designs for inclusion (e.g., randomised controlled trials, non-randomised controlled trials, before and after studies and interrupted time-series studies, analytical observational studies including prospective and retrospective cohort studies, case-control studies, analytical cross-sectional studies, descriptive observational study designs including case series, individual case reports and descriptive cross-sectional studies).

Qualitative studies were also considered that focused on qualitative data including, but not limited to, designs such as phenomenology, grounded theory, ethnography, qualitative description, action research and feminist research. In addition, systematic reviews that met the inclusion criteria were considered, depending on the research question. Editorials and opinion papers were also considered for inclusion in the scoping review.

A range of data were required to be as inclusive as possible due to the diverse nature of how research culture is reported and discussed in the public domain (and its associated parts in Open Access (OA), Equality, Diversity, and Inclusion (EDI) and career paths). Therefore, the review included published material from academic outputs (e.g., Journal articles, commentaries, editorials, perspectives, opinion letters) and from grey literature (e.g., reports, blogs, web-based articles, and newsletters including associated webpages of relevance).

### Search strategy

The search strategy aimed to locate both published and unpublished citations. An initial limited search of Medline and Web of Science (WoS) was undertaken to identify articles on the topic, to develop and pilot the search strategy. The text words contained in the titles and abstracts of relevant articles and reports, and the index terms used to describe the articles were used to develop a full search strategy. ^15^^,^^35^^–^^38^ The search strategy, including all identified keywords and index terms, were adapted for each included database and/or information source.

There were no study or language limits applied in the information retrieval process. The search strategy was limited from 2017 to 2022 but a preliminary search of citations during 2015-2022 was initially screened for relevance. Preliminary scoping and piloting of the search terms and strategies suggested that five years was sufficient for literature to be relevant, current, and broad (including relevant citations on the reporting of initiatives such as DORA, and any changes due to the COVID-19 pandemic). The review included UK and international literature (including grey literature, although see below for pragmatic restrictions for news items).

*Databases* **:** Six databases were searched (Medline, Engineering village, Scopus, JSTOR, ProQuest and WoS) during the period 29 April to 18 May 2022. A range of databases enabled the reviewers to capture several disciplines and to be as inclusive as possible.

*Grey literature searches **:*** A pragmatic systematic search was undertaken of the Lexis-Nexis Academic database concentrating on newspapers and news items. The scoping and piloting of the search terms in the database suggested that geographical exclusions were needed due to the scale of results from the searches. As part of discussions with team members as well as an experienced librarian, the results were filtered to only include news outlets and organisations based in UK, Europe, North America, and Australasia. To augment the news searches, purposive sampling of relevant research websites was documented in an Excel spreadsheet to record all platforms and webpages visited. The sampling of websites was drawn from discussions with team members as well as an experienced librarian. Examples of research websites explored include: The Conversation, Nature, Science, UK Research and Innovation (UKRI) and Research on Research Institute (RoRi). Relevant citations were identified from Wellcome Trust ^14^^,^^15^ as well as snowballing. The key references of the included articles and/or reports were screened for additional citations to be included as part of the overall screening process (including grey literature).

### Data extraction and evidence selection

Following the searches, all identified articles were collated and uploaded into Endnote version 20 ( *Clarivate Analytics, PA, USA,* *https://endnote.com/* ) (a free alternative is Mendeley: https://www.mendeley.com/) and duplicates were removed. Following a pilot test, all titles and abstracts were screened, by two independent reviewers for assessment against the inclusion criteria for the review using Rayyan. Potentially relevant articles were then retrieved for full extraction. At the full citation screening stage, reasons for exclusion were noted independently by both reviewers. Where the independent reviewer was unsure, the article was discussed, and a decision was made by consensus. Screening at both stages (title and abstract and full extraction) was piloted using Rayyan and labels were applied to categorise the focus of the articles based on four areas:

- Security (including career paths, career progression, stability contracts/careers, issues affecting early career researchers etc.)
- Wellbeing and equality of opportunity (including equality, diversity and inclusion, mental health, and wellbeing, bullying and harassment)
- Teamwork (including team science, recognition of broad contribution to research, incentives)
- Research quality and accountability (including research integrity, reproducibility, policy, and governance).

These focus areas were reported in the Wellcome Trust report and formed the basis of the current scoping review, to enable the University of Southampton to build on activity already undertaken, activity underway and enable alignment for future consideration. ^14^^,^^15^

**Figure 1.**
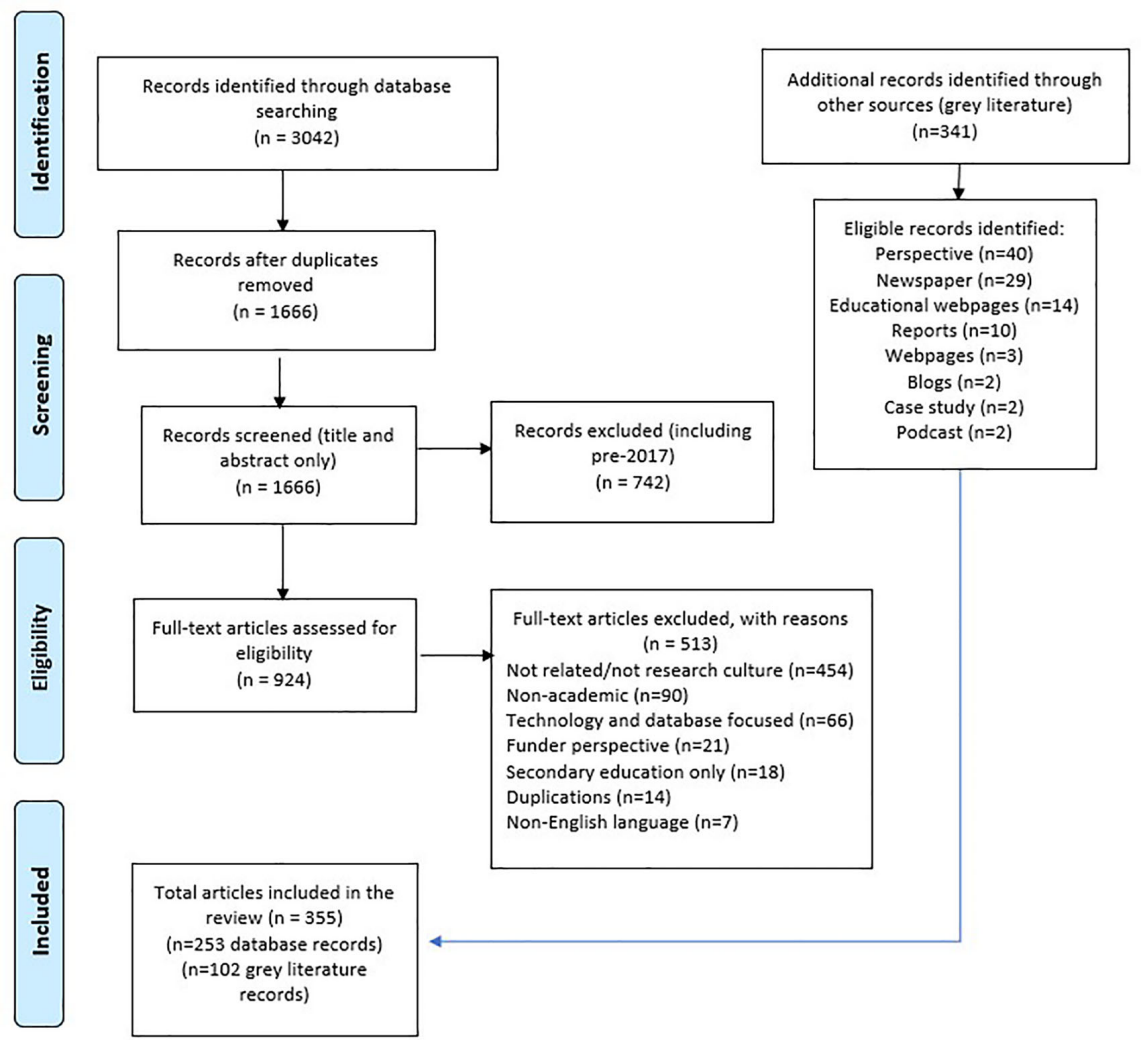
PRISMA flow diagram.

The list of included articles for full extraction were then exported to a Microsoft Excel spreadsheet using the labelling of articles from Rayyan (these categories were grouped together under the four focused areas). The results of the search and the study inclusion process are reported in full using the Preferred Reporting Items for Systematic Reviews and Meta-analyses extension for scoping reviews (PRISMA-ScR), including the flow diagram reported in Figure 1 *.*

Both reviewers extracted data from the full text articles using a data extraction tool developed by the reviewers to address the research question. This included the focus of the article, issues and/or problems reported in the article, solutions and/or recommendations provided in the article and details about whether the article related to more than one topic area.

No risk of bias or assessment on quality was conducted due to using a scoping review methodological approach. All the evidence was mapped and categorised into the four areas, which were discussed and agreed between team members at various stages of data extraction and during the write-up of the findings.

## Results

A total of 3,042 articles were retrieved from the six databases. With 1,376 duplications that were removed, 1666 titles and abstracts and 924 full text articles were assessed for eligibility. Of these 924 full text articles, 253 articles met the eligibility criteria for inclusion.

A total of 341 documents were retrieved (Lexis-Nexis) or identified across all the sources based on the titles. These were assessed for eligibility of which 102 met the criteria for inclusion.

Figure 1 provides a full account of the records of identification flow diagram, including the reasons for the excluded articles.

### Characteristics of the included studies

From the evidence there was a steady rise in the number of published articles over the last five years, with a notable increase from 2019. Table 1 shows that from the 253 included articles, there were 135 original research articles (this included qualitative and quantitative studies), 20 review articles (using a range of methodological review approaches), 86 perspective articles and 10 conference proceedings.

**Table 1.** Characteristics of the included studies.

Characteristics	N=253 (%) Databases	N=102 (%) Grey literature
*Areas of focus:* *		
Security	72	69
Wellbeing and equality of opportunity	52	50
Teamwork	64	40
Research quality and accountability	133	52
*Year of publication:*		
2017	20 (7.9)	12 (11.7)
2018	18 (7.1)	30 (29.4)
2019	47 (18.6)	15 (14.7)
2020	61 (24.1)	11 (10.8)
2021	85 (33.6)	27 (26.5)
2022 **	22 (8.7)	7 (6.9)
*Country:*		
UK	17 (6.7)	7 (6.9)
Europe	36 (14.2)	2 (1.9)
USA and Canada	71 (28.2)	4 (3.9)
International	73 (28.7)	11 (10.8)
NA	56 (22.2)	78 (76.5)
*Article type:*		
Journal – Original research (including panel discussions)	135 (53.5)	0
Journal – Review	20 (7.9)	0
Journal – Perspective ***	86 (33.9)	40 (39.3)
Conference proceeding	10 (3.9)	0
Book	2 (0.8)	0
Blog	0	2 (1.9)
Case study	0	2 (1.9)
Newspaper	0	29 (28.5)
Podcast	0	2 (1.9)
Report	0	10 (9.8)
Webpages/Educational webpages	0	17 (16.7)

* Note that some articles reported under more than one area of focus. The total number does not equal to the number of articles included in the scoping review.

** Jan-April inclusive, searches were conducted during April-May 2022.

*** Includes, editorial, commentaries, news features, correspondence, and perspective articles in journals.

The location of the study generation was captured for the included articles (based on location of the research and/or authors location). The included articles covered a global perspective with 71 articles from USA and Canada, 73 from international locations such as Africa (n=13), China (n=7), Australia (n=7) and Pakistan (n=4), 36 from Europe and 17 from the UK.

The grey literature provided 102 additional materials, 40 perspective articles reported in journals, 29 newspaper articles, 17 webpages (including educational webpages such as The Conversation: https://theconversation.com/uk) and including 10 reports. The remaining six were either a podcast, blog or case study. A majority of the grey literature material could not be grouped by location due to the nature of the material (76.5%, 78/102).

### Summarising the evidence

The evidence found in the database searches and grey literature was grouped according to the four focused areas, based on the key concepts developed during the full screening of the articles (based on the Wellcome Trust report). ^15^ Several included articles were relevant to more than one focus area, which showed the breadth of the topic but also how these areas are overlapping and mutually reinforcing. For example, evidence reported under security was also closely linked to wellbeing and equality of opportunity (especially for early career researchers (ECRs) and Science, Technology, Engineering, Mathematics and Medicine (STEMM)).

**Figure 2.**
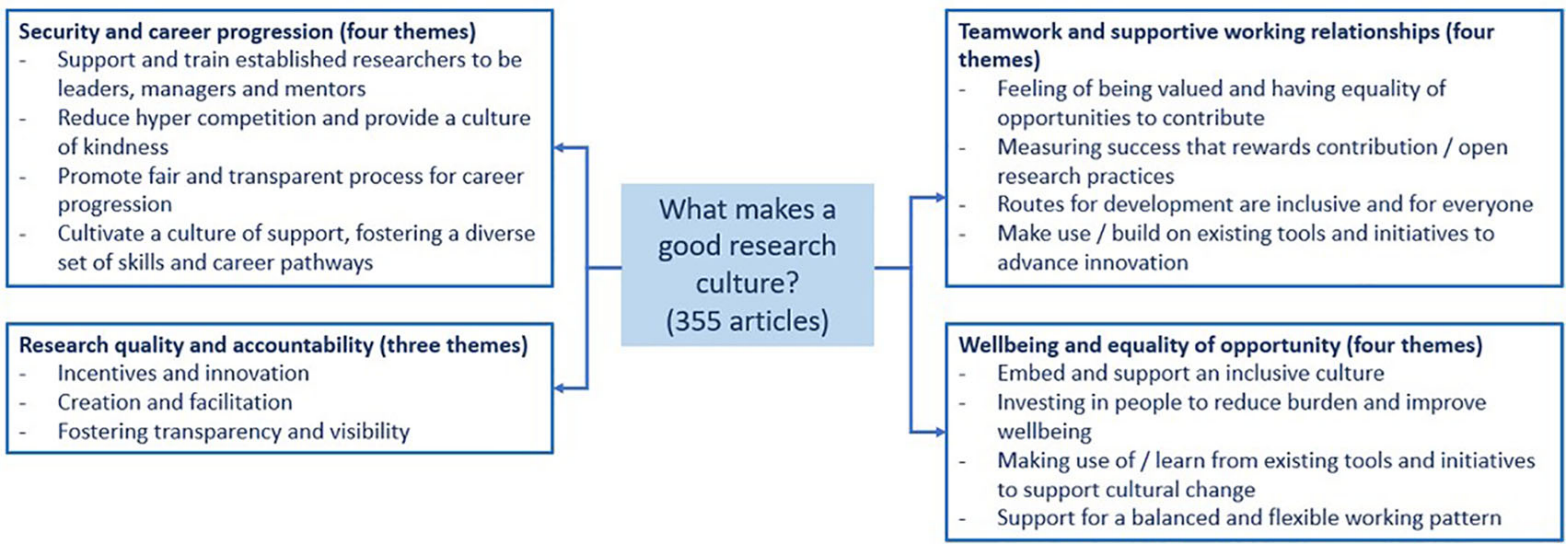
Summary of the evidence base on the four focused areas.

The sections below provide a summary of the evidence based on the four focused areas, with particular attention on security, wellbeing, equality of opportunity and teamwork, and research quality and accountability. ^14^^,^^15^ (see Figure 2) The quality or assessment of these initiatives were not explored as part of this scoping review and the key considerations arising from the evidence are not presented in order of priority.

### Security and career progression

From the evidence it was clear that there is a global drive to expose the challenges and barriers in academic research culture. It was evident that these factors were not exclusive to specific countries, disciplines, or research institutions and the challenge is at a system level. Concerns over job security, career progression and sustainability were particularly experienced by PhD students, ECRs and junior researchers from a range of academic environments. ^39^^–^^42^ The evidence reflects the range of research careers, roles, skills and expertise that are involved in research activity. Research Institutions often separate staff into job families such as research, education, technical, clinical, and managerial and professional roles titles reflecting different career pathways. ^17^^,^^43^^–^^45^

The evidence suggests there are concerns that research institutions are not managing career progression expectations or providing ways to develop and train staff, which can result in inequality between career types. ^17^^,^^43^^–^^45^ This can accumulate in feelings of failure by staff, or staff feeling pressured to be successful, which ultimately could promote unhealthy working practices such as excessive workloads and working long hours to meet expectations. ^46^^–^^48^ These issues affect all research institution staff but the evidence suggests they are particularly acute for several groups such as ECRs, STEMM, people on Fixed Term Contracts (FTC), people with caring responsibilities, and people with disabilities ^46^^–^^48^ (see Table 2 for a summary of the key considerations associated to security and career progression).

**Table 2.** Key concepts and statements associated to security and career progression.

Key themes	No	Statements	No. refs
**Support and train established researchers to be leaders, managers, and mentors**	1	Consider evaluating supervisors and mentor support by including impact statements of projects and career progression, including encouraging multiple career paths, benefits of collaboration across disciplinary boundaries and pooling scarce resources ^8^^,^^14^^,^^24^^,^^44^^–^^47^^,^^51^^–^^83^	40
	2	Provide all staff with access to flexible approaches and methods to mentoring and peer coaching schemes, enabling self-learning, innovation and productivity ^6^^,^^17^^,^^46^^,^^47^^,^^52^^,^^53^^,^^55^^,^^56^^,^^58^^,^^61^^,^^62^^,^^64^^,^^66^^–^^69^^,^^71^^–^^73^^,^^75^^,^^76^^,^^78^^,^^81^^,^^82^^,^^84^^–^^98^	35
	3	Consider feedback initiatives to support continued professional development for supervisors and team leaders or collate benchmarking data focused on the culture of the research team between supervisor-student ^23^^,^^24^^,^^42^^,^^43^^,^^48^^,^^54^^,^^57^^,^^59^^,^^60^^,^^62^^,^^64^^,^^66^^,^^70^^,^^80^^,^^92^^,^^98^^–^^111^	30
	4	Explore opportunities for leadership and management training (including project management) for all staff, levels, stages, and position within academia) ^24^^,^^43^^,^^54^^,^^57^^,^^60^^,^^62^^,^^64^^,^^66^^,^^67^^,^^70^^,^^90^^,^^95^^,^^96^^,^^100^^,^^103^^,^^104^^,^^107^^,^^112^^–^^121^	27
	5	Provide those who supervise PhDs, counsel researchers (including ECRs/junior staff) with clear guidelines on best practice and mechanisms for support (including benefits of networking), encouraging an open mind about career progression ^26^^,^^45^^,^^51^^,^^52^^,^^57^^,^^59^^–^^62^^,^^66^^,^^69^^,^^74^^,^^79^^,^^81^^,^^83^^,^^85^^,^^104^^,^^114^^,^^122^^–^^129^	26
	6	Seek ways to reduce the administrative burden for those involved in leading and managing research, including innovative tools to support meaningful networking connections (e.g., MyNRMN, National Research Mentoring Network, Kaupapa Māori Frameworks) ^8^^,^^24^^,^^46^^,^^53^^,^^55^^,^^61^^,^^62^^,^^66^^,^^67^^,^^71^^,^^73^^,^^78^^,^^81^^,^^82^^,^^85^^,^^90^^,^^92^^,^^107^^,^^126^^,^^129^	21
	7	Explore ways to support staff with line management skills including appraisal and inclusive management practices and motivate managers to prioritise these duties, including upskilling of staff ^43^^,^^54^^,^^69^^,^^118^^,^^125^^,^^130^	6
**Reduce hyper competition and provide a culture of kindness**	1	Recognise and incentivize all staff for developing equitable practices and partnerships (e.g., capacity building with Low Middle Income Countries (LMICs) as collaborators and beneficiaries) and focus less on publication numbers and citations (including grant awards) ^4^^,^^17^^,^^37^^,^^41^^,^^57^^,^^68^^,^^70^^,^^80^^,^^94^^,^^102^^–^^106^^,^^108^^,^^109^^,^^116^^,^^117^^,^^130^^–^^139^	28
	2	Provide regular opportunities for informal, open, safe and honest conversations (and how to optimise the role of networking), including environmental tensions between research, education, teaching etc. ^42^^,^^47^^,^^48^^,^^52^^,^^57^^,^^61^^,^^63^^,^^67^^,^^69^^,^^81^^,^^95^^,^^104^^,^^113^^,^^122^^,^^125^^,^^132^^,^^136^^,^^137^^,^^140^^–^^144^	24
	3	Consider ways to demonstrate support for other researchers to secure funding as part of progression (and review current reward systems), ensuring education, teaching and research are equally prioritised ^37^^,^^48^^,^^55^^,^^60^^,^^61^^,^^88^^,^^94^^,^^96^^,^^114^^,^^124^^,^^132^^,^^137^^,^^140^^–^^143^^,^^145^	17
	4	Encourage staff to provide positive feedback and praise to each other, making the working environment friendly, productive and conducive for learning ^6^^,^^48^^,^^62^^,^^63^^,^^84^^,^^86^^–^^88^^,^^91^^,^^95^^,^^97^^,^^98^^,^^139^^,^^141^^,^^145^^–^^147^	17
	5	Raise awareness on the value of sharing science outreach goals, promoting mutual learning for all (including academic induction and orientation practices) to help with retention and progression ^17^^,^^40^^,^^54^^,^^55^^,^^59^^,^^63^^,^^66^^,^^68^^,^^95^^,^^115^^,^^122^^,^^137^^,^^138^^,^^143^^,^^144^^,^^148^^,^^149^	17
	6	Provide opportunities for shared learning and to develop from failure in funding applications and publishing, avoiding the stresses of perceived failure ^47^^,^^59^^,^^68^^,^^114^^,^^122^^,^^130^^,^^150^	7
**Promote fair and transparent process for career progression**	1	Ensure greater alignment between individual and institutional values that encourages teamwork (team science), collective leadership and shared decision-making, building a more supportive academic culture, and facilitating institutional and departmental recognition in faculty career development ^17^^,^^23^^,^^24^^,^^26^^,^^37^^,^^41^^,^^43^^–^^45^^,^^47^^,^^48^^,^^51^^,^^54^^,^^55^^,^^57^^,^^60^^,^^62^^,^^63^^,^^65^^–^^69^^,^^72^^,^^74^^,^^79^^,^^83^^,^^87^^,^^89^^,^^91^^,^^93^^,^^98^^–^^107^^,^^109^^,^^110^^,^^112^^,^^114^^,^^117^^,^^123^^,^^125^^,^^130^^,^^133^^,^^134^^,^^137^^,^^138^^,^^140^^,^^141^^,^^143^^,^^149^^,^^151^^–^^154^	61
	2	Consider approaches to award and recognition of performance that is not solely based on academic publications that is fair and transparent for all staff, differentiating key performance areas, as well as the workload of academics in various career stages and positions ^4^^,^^17^^,^^23^^,^^45^^,^^47^^,^^52^^,^^54^^,^^55^^,^^57^^,^^63^^,^^68^^,^^79^^,^^101^^,^^103^^,^^114^^,^^116^^,^^123^^,^^125^^,^^132^^,^^135^^–^^137^^,^^139^^,^^142^^,^^144^^,^^145^^,^^151^^,^^152^^,^^154^	29
	3	Raise awareness about using research metrics responsibly and appropriately. For example, do not use journal-based metrics, such as Journal Impact Factors, as a surrogate measure of the quality of individual research articles, to assess an individual scientist’s contributions, or in hiring and promotion (flaws in the current awards system not promoting research) ^3^^,^^4^^,^^20^^,^^26^^,^^37^^,^^50^^,^^56^^,^^61^^,^^68^^,^^84^^,^^124^^,^^126^^,^^139^^,^^155^^,^^156^	15
	4	Consider and optimise how academic CVs are used. Provide instructions for researchers and evaluators; prioritise actual achievements; focus on recent achievements, relevant activities and outputs; acknowledge the broad range of contributions; balance and control incentives; use academic age not biological age; encourage narratives; and use metrics cautiously (Open Researcher and Contributor ID organization (ORCID) as a spin-off from ORCID’s Reducing Burden and Improving Transparency (ORBIT) project to foster exchange and pool expertise, to optimise the responsible use of contributions and metrics) ^3^^,^^4^^,^^20^^,^^26^^,^^50^^,^^55^^,^^56^^,^^84^^,^^114^^,^^126^^,^^155^^,^^156^	12
**Cultivate a culture of support that fosters a diverse set of skills and career pathways**	1	Raise awareness of the issues surrounding research culture amongst ECRs so they can contribute to the University, their departments or research teams by: facilitating membership of formal networks, provide opportunities to connect with colleagues for social and work-related activities, and review policies around office attendance that support integration and innovation ^6^^,^^23^^,^^26^^,^^40^^,^^41^^,^^46^^,^^48^^,^^54^^–^^57^^,^^61^^,^^63^^,^^69^^,^^87^^–^^89^^,^^93^^,^^98^^,^^99^^,^^101^^,^^104^^–^^106^^,^^109^^,^^119^^,^^126^^–^^128^^,^^137^^,^^149^^,^^150^^,^^154^	34
	2	Consider ways to embed ‘career optimism’ to teach, prepare and respect the diversity of career pathways of PhD students/early career researchers within and beyond the University (and finding your research niche) ^17^^,^^23^^,^^42^^,^^44^^–^^46^^,^^51^^,^^52^^,^^54^^,^^55^^,^^59^^,^^63^^,^^83^^,^^96^^,^^101^^,^^102^^,^^111^^,^^115^^,^^119^^,^^123^^,^^127^^,^^128^^,^^142^^–^^144^^,^^151^^,^^153^^,^^154^^,^^157^	29
	3	Provide opportunities to encourage ECRs to join and engage in conversations that affect them, such as research assessment, career progression, awards system (including sabbatical leave) ^3^^,^^6^^,^^26^^,^^44^^,^^54^^,^^55^^,^^60^^,^^61^^,^^63^^,^^74^^,^^83^^,^^88^^,^^96^^,^^101^^,^^104^^,^^105^^,^^115^^,^^117^^,^^119^^,^^124^^,^^126^^–^^128^^,^^145^^,^^149^^,^^151^^,^^155^^–^^157^	29
	4	Recognise and value the diverse skill-set of research managerial and technical staff and provide opportunities for them to host and supervise researchers, apply for research grants and undertake research promoting the benefits of collaboration rather than competitive research culture ^4^^,^^8^^,^^17^^,^^37^^,^^41^^,^^44^^,^^45^^,^^59^^,^^63^^,^^68^^,^^80^^,^^91^^,^^94^^,^^95^^,^^104^^,^^106^^,^^109^^,^^110^^,^^118^^,^^142^^,^^147^^,^^149^^,^^153^^,^^154^^,^^157^^,^^158^	27
	5	Offer opportunities to build hybrid careers by offering different and multiple pathways and opportunities in research, including alignment with the private sector and employment outside of academia for when long-term academic employment is not viable ^6^^,^^45^^,^^52^^,^^55^^,^^65^^,^^74^^,^^79^^,^^83^^,^^102^^,^^113^^,^^115^^,^^117^^,^^122^^,^^124^^,^^142^^,^^149^^,^^151^^,^^153^^,^^154^	19
	6	Consider how to develop a career development mindset that supports people in all aspects of research not just research projects, therefore attracting a diverse workforce and provide greater career stability (e.g., type of contract) ^44^^,^^45^^,^^52^^–^^55^^,^^63^^,^^73^^,^^83^^,^^100^^,^^101^^,^^103^^,^^125^^,^^145^^,^^151^^,^^154^^,^^157^^,^^159^	18
	7	Offer or provide opportunities for writing retreats, boot camps, away days and mentoring including cross disciplinary training, enabling informed decisions such as choosing your own mentor ^17^^,^^46^^,^^52^^,^^53^^,^^58^^,^^64^^,^^69^^,^^71^^–^^73^^,^^75^^,^^76^^,^^82^^,^^89^^,^^96^^,^^117^^,^^160^	17
	8	Consider how those in research management and technical roles (including librarians) have adequate routes to continued professional development through inhouse or formal training (examples from University of California Curation Center, part of the CDL, and the Digital Curation Centre in Edinburgh, UK ^)(40,43,70,95,96,108,113,117,119-121,142,147,149,153,158)^	16
	9	Explore ways to implement a ‘culture of structure’ for graduate students where expectations are clear and students have contact with multiple faculty members, including focus on sources that support all students, faculty, and staff ^23^^,^^40^^,^^42^^,^^45^^,^^55^^,^^59^^,^^91^^,^^92^^,^^102^^,^^110^^,^^111^^,^^122^^,^^144^^,^^152^^,^^154^	15

The review suggests that the problem is reinforced by a culture where researchers are incentivized to produce many funding applications and academic publications where high rejection rates. ^47^ A related issue identified was the risk of evaluating academic performance based on the use of inadequate proxies (e.g., publication productivity, impact factor and citations). ^3^^,^^49^ Evidence suggests that this can result in a lack of workload oversight, a culture discouraging of appreciation, that in turn makes researchers feel pressured to be successful, often resulting in a significant amount of time in pursuit of success at the cost of their wellbeing. ^42^^,^^46^^,^^47^^,^^49^^–^^51^

The concept of research culture and job security was broad and included (but is not limited to) career paths; stability of contracts and careers; and issues affecting ECRs and students. ^57^^,^^141^^,^^145^ A wide range of initiatives were explored in the literature covering a broad spectrum of factors (as detailed in Table 2). The evidence showed how, where, and why changes are needed to establish a global cultural change to the research ecosystem to enable fair and transparent progression for all research staff ^99^^,^^104^^,^^161^; cultivate a culture that fosters diversity across career pathways ^17^^,^^55^ and; initiate deeper integration of knowledge to ensure institutional stability. ^57^^,^^153^

The evidence suggests that offering potential solutions or supportive actions for academic institutions and the research community may enhance and stabilise career paths, particularly those in the early career stage, including those in technical and managerial roles. Although these solutions and supportive actions are by no way exhaustive, they do provide a summary of the range of factors that could go some way in promoting a better research culture. ^54^^,^^138^^,^^154^^,^^162^

### Wellbeing and equality of opportunity

A key consideration from the evidence suggests that there are disparities across the research ecosystem, which are in turn influencing individuals’ wellbeing. ^39^^,^^40^^,^^43^^,^^70^^,^^92^^,^^96^^,^^99^^,^^118^^,^^163^^–^^166^ The impact of these disparities are preventing or slowing down initiatives to seek for a cultural change in the academic environment. Although there has been some progress, the evidence suggests progress is slow and continues to be a challenge, especially for under-represented groups. ^166^^–^^169^ The disparities highlighted in the literature suggests that under-represented groups are less likely to be promoted or receive funding, and have a higher risk of decreased well-being. ^18^^,^^64^^,^^83^^,^^89^^,^^170^^–^^179^ However, as Lee (2022) pointed out, although underrepresented groups and junior staff are more likely to experience these challenges, including microaggression (and being victimised regardless of their role or position), anyone can, at some point in their academic career experience some form of microaggression (e.g., bullying, patronage power, exploitation, discrimination). ^20^ This also extends to the notion of imposter syndrome as noted by Hagan (2020). ^169^ Moreover, the way disciplines are taught at university means that curricula focusing on traditional perspectives may not be inclusive to everyone. ^123^^,^^176^

The review revealed that the risks associated with a lack of diversity and inclusion often result in individuals leaving academia, low job satisfaction, increased stress, burnout and mental health problems, and decreased productivity. ^18^^,^^44^^,^^74^ The evidence suggests that these issues have an impact not only at the institutional level such as having a lack of diversity in organisational leaderships, ^73^^,^^97^^,^^180^ but also for individuals, leading to a lack of role models and peer mentors ^163^ skills shortages in particular disciplines, sectors and roles, ^44^^,^^92^^,^^181^ and drives off talent. ^8^ Moreover, the increasing demands of heavy workloads and the risk of perpetuating a culture of academic rejection can impact an individual’s wellbeing. ^18^^,^^46^^,^^47^^,^^106^^,^^182^ Table 3 provides a summary of the key considerations associated to wellbeing and equality of opportunity.

**Table 3.** Key concepts and statements associated to wellbeing and equality of opportunity.

Key themes	No	Statements	No. refs
**Embed and support an inclusive culture**	1	Create a climate for diversity and inclusivity by working collectively to reduce attitudes of hostility and competition that are pervasive, including among STEM fields (e.g., SciComm, diversity programs), including combating the barriers to inviting diverse speakers and self-nomination ^6^^,^^44^^,^^69^^,^^73^^,^^78^^,^^79^^,^^81^^–^^83^^,^^89^^,^^106^^,^^109^^,^^111^^,^^120^^,^^123^^,^^129^^,^^146^^,^^147^^,^^156^^,^^170^^,^^171^^,^^173^^,^^175^^,^^176^^,^^183^^–^^202^	43
	2	Lead with data by moving from expert opinion and commentary on effective measures for advancing Diversity Equity and Inclusion (DEI) to objective, validated, and evidence-based research and evaluation ^6^^,^^18^^,^^73^^,^^78^^,^^79^^,^^81^^–^^83^^,^^88^^,^^89^^,^^98^^,^^106^^,^^109^^,^^111^^,^^120^^,^^129^^,^^156^^,^^166^^,^^167^^,^^170^^,^^173^^,^^175^^–^^177^^,^^189^^–^^191^^,^^193^^–^^195^^,^^198^^–^^207^	41
	3	Enable honest conversations around the complexities, challenges and barriers to achieving diversity in leadership ^6^^,^^73^^,^^78^^,^^79^^,^^81^^–^^83^^,^^89^^,^^92^^,^^98^^,^^106^^,^^109^^,^^111^^,^^120^^,^^123^^,^^129^^,^^156^^,^^165^^,^^167^^,^^175^^,^^176^^,^^184^^–^^186^^,^^188^^,^^193^^,^^195^^,^^197^^–^^205^	36
	4	Diversify visible reporting routes to encourage institutions to move away from performative actions and acknowledge that institutional factors play a role in improving mental health for individuals (e.g., ‘Me Too’ movement, #STEMToo social media hashtag to share stories) ^6^^,^^18^^,^^20^^,^^42^^,^^46^^,^^51^^,^^69^^,^^74^^,^^75^^,^^79^^,^^80^^,^^89^^,^^91^^,^^97^^,^^101^^,^^107^^,^^111^^,^^146^^,^^147^^,^^171^^,^^179^^,^^189^^–^^191^^,^^197^^,^^202^^,^^208^^,^^209^	28
	5	Encourage and support staff and students to build support groups, to reach out for help, to talk openly about mental health, and to ask how others are doing ^6^^,^^20^^,^^42^^,^^46^^,^^51^^,^^74^^,^^75^^,^^79^^,^^80^^,^^89^^,^^91^^,^^96^^,^^97^^,^^101^^,^^107^^,^^111^^,^^120^^,^^123^^,^^173^^,^^198^^,^^202^^,^^209^	22
	6	Use institution-specific data to drive changes in policy and programming to improve the social culture and climate, including shift institutional practice in a context-specific way ^61^^,^^89^^,^^98^^,^^101^^,^^109^^,^^162^^,^^167^^,^^169^^,^^170^^,^^177^^,^^179^^,^^184^^,^^186^^,^^197^^,^^210^^,^^211^	16
	7	Create greater emphasis on cultural competency, to enable the ability to understand, honor, appreciate, and respect the values, beliefs, attitudes, and behaviors of those from cultures different to our own ^89^^,^^98^^,^^101^^,^^109^^,^^167^^,^^169^^,^^170^^,^^173^^,^^183^^,^^195^^,^^212^	11
	8	Enable conversations that shine a light on power imbalances within academia through initiatives (e.g., “Me Too” Movement) ^18^^,^^122^^,^^165^^,^^167^^,^^169^^,^^170^^,^^177^^,^^195^^,^^197^^,^^204^^,^^211^	11
	9	Explore avenues which will help to identify how disciplines could be taught through a more inclusive and ethical lens, ensuring that socio-economic data on employees is collected and monitored (as recommended by the Social Mobility Commission) ^123^^,^^189^^–^^192^^,^^194^^,^^195^	7
	10	Improve professional workplace mental health and access to services/support for mental health and ensure that use of such services does not stigmatize. In addition, invest to improve mental health literacy across the institution, supporting those who provide assistance and training in mental health, particularly post COVID ^18^^,^^46^^,^^51^^,^^96^	4
**Investing in people to reduce burden and improve wellbeing**	1	Train leaders, principal investigators, staff and students in mental health and diversity, and dignity and respect ^6^^,^^20^^,^^42^^,^^46^^,^^51^^,^^74^^,^^75^^,^^78^^–^^82^^,^^89^^,^^91^^,^^97^^,^^101^^,^^107^^,^^109^^,^^120^^,^^123^^,^^129^^,^^156^^,^^164^^,^^175^^,^^176^^,^^184^^,^^185^^,^^187^^,^^190^^–^^193^^,^^198^^–^^202^^,^^205^^,^^209^	42
	2	Enable access to childcare near or on campus, extend paid maternity/parental leaves, parttime options for work, a career pause during children’s formative childrearing years, greater access to administrative and research support, and the fundamental recognition of family status in academic policies and practices ^6^^,^^20^^,^^42^^,^^46^^,^^51^^,^^73^^–^^75^^,^^79^^,^^80^^,^^83^^,^^89^^,^^91^^,^^96^^,^^97^^,^^101^^,^^107^^,^^111^^,^^122^^,^^146^^,^^147^^,^^165^^,^^167^^,^^177^^,^^179^^,^^196^^,^^203^^,^^209^^,^^213^	29
	3	Provide or maximise mentorship, sponsorship and collaboration between academics at all stages of their career ^44^^,^^46^^,^^47^^,^^61^^,^^64^^,^^69^^,^^73^^,^^89^^,^^98^^,^^101^^,^^109^^,^^162^^–^^165^^,^^172^^,^^184^^,^^193^^,^^196^^,^^197^^,^^199^^,^^202^^,^^212^^,^^214^	27
	4	Consider courses aimed at underrepresented groups to improve confidence, assertiveness and to manage negative influences, such as imposter syndrome; empower staff through participation in decision-making/problem solving ^118^^,^^162^^,^^173^^,^^179^^,^^187^^,^^197^^,^^199^^,^^210^^–^^212^^,^^214^	11
	5	Consider pre-assessing research skills so that different types of mentor-mentee matching strategies can be formed in as many areas as needed, which can help new investigators, early-stage investigators and underrepresented minorities ^47^^,^^61^^,^^64^^,^^69^^,^^163^^,^^184^^,^^193^^,^^203^^,^^212^	9
	6	Reward or emphasize collaboration over competition ^44^^,^^80^^,^^89^^,^^98^^,^^101^^,^^109^^,^^215^	7
**Making use of and learning from existing tools and initiatives to support cultural change**	1	Consider adopting an inclusive and shared leadership model such as Networked Improvement Community (NIC) which focuses on shared leadership, inclusive practices in different contexts (e.g., for STEM, establishing a culture of equity and engagement) to strengthen infrastructure at local levels ^69^^,^^78^^,^^89^^,^^96^^,^^98^^,^^101^^,^^109^^,^^146^^,^^147^^,^^164^^,^^165^^,^^169^^,^^171^^,^^173^^,^^177^^,^^179^^,^^183^^,^^184^^,^^189^^,^^194^^,^^197^^,^^204^^–^^206^^,^^215^^,^^216^	26
	2	Encourage/signpost staff to peer groups to enable and encourage networking and shared understanding including critically reflect on cultural identity(e.g., Blackett Lab Family, Black Heroes of Mathematics, Africans in STEM) ^69^^,^^122^^,^^146^^,^^164^^,^^166^^,^^170^^,^^171^^,^^175^^,^^207^^,^^214^^,^^217^	11
	3	Implement and encourage staff development opportunities (e.g., StellarHE) that are inclusive to and for everyone, regardless of characteristics, career stage or job role, including Learn from networks and initiatives such as the National Research Mentoring Network (NRMN), Athena Swan (UK based), sign up to UK’s Concordat to Support the Career Development of Researchers ^53^^,^^69^^,^^123^^,^^162^^,^^163^^,^^179^^,^^189^^,^^194^^,^^197^^,^^203^	10
	4	Consider adopting PRESS, an evidence-based framework for achieving racial equity; as well as using well-designed metrics that can help to manage discrimination ‘blind spots’ and encourage a 'sense of belonging' (e.g., Challenged Sense of Belonging Scale) ^166^^,^^169^^,^^173^^,^^180^^,^^204^^–^^206^^,^^217^	8
	5	Seek to safeguard the physical and emotional well-being of PEER trainees, by equipping STEM allies with tools to combat discrimination (e.g., Allyship for PEER trainees (Persons Excluded from science because of Ethnicity and Race (PEERs)) ^69^^,^^122^^,^^146^^,^^166^^,^^169^^,^^171^^,^^180^^,^^214^	8
	6	Increase uptake of digital tools and inclusion-sensitive pedagogy to support equal participation in Higher Education programmes, including promotional materials and promote open knowledge institutions (OKIs) in diversity, communications and coordination, support opportunities for virtual conferences to increase access for researcher participation in training, symposia, and conferences ^162^^,^^168^^,^^180^^,^^185^^,^^187^^,^^194^^,^^208^	7
	7	Consider the use of Authentic Interrogation, Acknowledgment, and Accountability that requires SciCommers to explicitly articulate the ways in which STEM and SciComm have been used as systems of oppression ^69^^,^^146^^,^^169^^,^^171^^,^^183^	5
**Support for a balanced and flexible working pattern**	1	Ensure staff contracts can accommodate better pathways for flexible working so there are no unintended consequences on career for focusing on care-giver responsibilities or changing circumstances ^69^^,^^78^^,^^92^^,^^96^^,^^118^^,^^122^^,^^146^^,^^147^^,^^162^^,^^165^^,^^167^^,^^169^^,^^177^^,^^179^^,^^196^^,^^203^^,^^213^	17
	2	Put in place options that help staff to return to work after a period of absence to improve transition back to work and promote life-work balance (for students and staff) ^18^^,^^69^^,^^96^^,^^118^^,^^120^^,^^123^^,^^146^^,^^162^^,^^165^^,^^167^^,^^177^^,^^196^^,^^198^^,^^203^	14
	3	Commit to the ring-fencing of research-time and ensure researchers confirm time against other duties (i.e., teaching, administration, marking and preparation), including leaders model healthy working practices ^46^^,^^48^^,^^78^^,^^122^^,^^146^^,^^162^^,^^212^	7
	4	Include working hours as a standing item on appraisals and manage expectations around working hours, breaks and holidays to reduce excessive working hours, including use more inclusive job descriptions in hiring processes ^46^^,^^48^^,^^96^^,^^122^^,^^156^	5

The review illustrates that over the last three years, the COVID-19 pandemic has highlighted some important challenges to this already highly pressured working environment, with mixed effects. ^18^^,^^44^^,^^74^ Although, the pandemic has brought about challenges to the research workforce, there is also emerging evidence over the last two years showing the potential benefits and opportunities as a result of COVID-19. ^18^^,^^44^^,^^64^^,^^73^^,^^74^^,^^97^^,^^177^^,^^194^ For example, use of online platforms for training and teaching purposes has opened up opportunities to bring together specific communities and countries. The pandemic also initiated ‘kindness in research’ where empathy replaced the usual expectations on work-life balance. ^73^^,^^97^^,^^180^ The review also identified clear efforts to improve and raise awareness of the need for academia to embrace the EDI agenda through several initiatives, movements, and policy implementations (as detailed in Table 3). Most prominently, focusing on efforts to improve individuals’ opportunities through networking, collaborations, mentoring and peer-to-peer support, balancing career, and family aspirations can help to guide inclusivity and strengthen infrastructure and local capacity. ^171^^,^^177^^,^^183^^,^^205^

### Teamwork and supportive working relationships

Collaboration, openness, and transparency were highlighted in the evidence as key indicators of success for driving forward a positive cultural change. However, the emerging evidence suggested that perverse incentives within the research ecosystem, a lack of training, opportunity, support, and infrastructure can undermine ambitions for change, which is further hampered by researcher perceptions. ^4^^,^^24^^,^^60^^,^^154^^,^^181^^,^^218^^,^^219^

The evidence pointed to a range of barriers that have repercussions on the notion of teamwork, such as the ongoing tradition of first and last authors taking most or all of the credit for the work, ^220^ the use of ‘gift authorship’ to enhance research publication of academics with poor research performance, ^5^ and the pressures to have global impact on the scientific community through high-quality scientific writing. ^164^ Researchers are incentivized to attain research excellence, which can often result in hyper-competitiveness and unfair working practices. ^4^^,^^19^^,^^24^^,^^50^^,^^59^^,^^88^^,^^89^^,^^206^ The evidence further suggested that the Research Excellence Framework (REF) in the UK has been criticised as promoting competition between departmental colleagues rather than collaboration due to criteria on contributors and the increased demand on publications. ^49^^,^^89^

These practices can have a detrimental impact for the promotion of research integrity and team science, especially for ECRs. Research careers encompass a range of roles, skills, and expertise but as the evidence suggests this is not universal, with some universities separating research active staff from colleagues such as research managers, technicians, administrators, and librarians, some of whom have research-level qualifications and experience. ^41^^,^^99^^,^^158^ Separation in this way could lead to inequality of how staff are included (or not), trained, mentored and perceived by fellow colleagues (see Table 4 for a summary of the key considerations associated to teamwork and supportive working relationships).

**Table 4.** Key concepts and statements associated to Teamwork and supportive working relationships.

Key themes	No	Statements	No. refs
**Everyone feeling valued and having equality of opportunities to contribute**	1	Encourage faculties to support collaborations and networks that provide a sense of mutual support and culture of team effort rather than individual competition, through interactive learning environments and faculty members as supporters and mentors ^5^^,^^19^^,^^23^^,^^26^^,^^41^^,^^43^^,^^45^^,^^46^^,^^49^^,^^59^^,^^60^^,^^69^^,^^72^^,^^73^^,^^87^^–^^89^^,^^92^^,^^95^^,^^98^^,^^99^^,^^101^^,^^107^^,^^109^^,^^130^^,^^141^^,^^164^^,^^172^^,^^177^^,^^178^^,^^181^^,^^193^^,^^206^^,^^212^^,^^220^^–^^236^	51
	2	Consider incentives and mechanisms to share open data and empower multi-disciplinary teams to reuse data, and adopt incentives that are transparent across funding agencies, journals and institutions (including replication research) ^23^^,^^26^^,^^41^^,^^43^^,^^48^^,^^49^^,^^87^^,^^89^^,^^98^^,^^99^^,^^101^^,^^109^^,^^141^^,^^154^^,^^215^^,^^218^^,^^220^^,^^232^^,^^233^^,^^235^^–^^240^	24
	3	Encourage transformative interdisciplinary research to diversify teams, deepen integration of knowledge and move beyond separate disciplinary research ^19^^,^^23^^,^^26^^,^^41^^,^^46^^,^^49^^,^^57^^,^^69^^,^^73^^,^^87^^–^^89^^,^^101^^,^^109^^,^^164^^,^^206^^,^^220^^,^^222^^,^^224^^,^^235^^,^^236^^,^^241^^,^^242^	23
	4	Bring researchers together under a common goal to address specific research issues through a challenge-led (problem-led) research approach (including the health of labs) ^19^^,^^45^^,^^46^^,^^57^^,^^69^^,^^73^^,^^88^^,^^89^^,^^92^^,^^98^^,^^101^^,^^158^^,^^178^^,^^224^^,^^229^^,^^239^^,^^241^	17
	5	Provide greater opportunities and capacity for technical and library staff to improve their own research skills through networking, collaborative partnerships and being contributors to research, including raising accessibility for multi-disciplinary teams and interaction ^45^^,^^90^^,^^95^^,^^107^^,^^130^^,^^158^^,^^215^^,^^224^^,^^234^^,^^243^^–^^245^	12
	6	Ensure that those in research management and technical roles have adequate routes to continued professional development through inhouse or formal training (including ethical considerations and issues) ^95^^,^^99^^,^^152^^,^^158^^,^^178^^,^^224^^,^^227^^,^^244^^,^^246^^–^^249^	12
	7	Recognise and value the diverse skillset of research management and technical staff (and early career researchers), and provide opportunities for them to host and supervise researchers, apply for research grants and undertake research ^41^^,^^45^^,^^59^^,^^95^^,^^158^^,^^227^^,^^231^^,^^239^^,^^246^	9
**Measuring success that rewards contribution and open research practices**	1	Ensure that the term research excellence is understood and qualified within assessment processes to minimise opportunities to reward individualism at the expense of the collaborative, and create environments that assess the performance of the collective rather than only individuals (e.g., performance-based research funding systems PBRFS, and productivity) ^3^^,^^5^^,^^6^^,^^8^^,^^21^^,^^23^^,^^26^^,^^50^^,^^57^^,^^84^^,^^87^^–^^89^^,^^91^^,^^92^^,^^94^^,^^97^^,^^98^^,^^107^^,^^109^^,^^130^^,^^139^^,^^153^^,^^158^^,^^212^^,^^221^^,^^223^^,^^227^^,^^233^^–^^236^^,^^242^^,^^250^^–^^255^	39
	2	Reward multidisciplinary work through separate evaluation structures to encourage team science initiatives (consider including data sharing and collegiality as part of employment evaluation criteria) ^23^^,^^24^^,^^26^^,^^41^^,^^43^^,^^49^^,^^87^^,^^89^^,^^98^^,^^99^^,^^101^^,^^109^^,^^154^^,^^164^^,^^206^^,^^215^^,^^220^^,^^222^^,^^226^^,^^235^^,^^236^^,^^242^	22
	3	Review how research is recognised, incentivised and rewarded (subjective and objective measures of quantity, quality and impact), including Key Performance Indicators (KPIs) and whether monitoring systems are contributing to optimum solutions (including financial support and elevating barriers) ^3^^,^^8^^,^^23^^,^^26^^,^^50^^,^^84^^,^^92^^,^^98^^,^^109^^,^^178^^,^^217^^,^^221^^,^^232^^,^^237^^,^^239^^,^^241^^,^^243^^,^^252^^,^^254^	19
	4	Review, consider and evaluate the value, role, and purpose of incentives. Consider questions such as ‘Do they foster scientific knowledge?’ and ‘Are large collaboratives, open science practices innovative enough?’ ^8^^,^^19^^,^^23^^,^^24^^,^^57^^,^^98^^,^^107^^,^^108^^,^^130^^,^^164^^,^^212^^,^^218^^,^^237^^,^^250^	14
	5	Reward credible research practices that are addressing problems with credibility, rigour, and reproducibility in grant guidelines (e.g., incorporating Registered Reports in two stage funding model). Seek to encourage practice across publishers and institutions to not disadvantage researchers who engage in open practices (consider frameworks to improve quality publication practices (QPPs)) ^8^^,^^23^^,^^24^^,^^45^^,^^98^^,^^108^^,^^227^^,^^233^^,^^237^^,^^240^^,^^247^^,^^250^^,^^256^	13
	6	Consider ‘human-oriented’ knowledge practices over ‘output-oriented’ practices so that researchers and educators are evaluated based on value, quality and contribution, including early career researchers ^4^^,^^45^^,^^145^^,^^177^^,^^193^^,^^221^^,^^231^^,^^241^^,^^247^	9
**Ensure routes for development are inclusive and for everyone (regardless of position, role or discipline)**	1	Develop and reward cross-disciplinary training and mentoring aligned with development of on the job skills to promote interdisciplinary insight ^19^^,^^46^^,^^60^^,^^69^^,^^72^^,^^73^^,^^88^^–^^90^^,^^92^^,^^95^^,^^98^^,^^101^^,^^152^^,^^154^^,^^172^^,^^181^^,^^193^^,^^217^^,^^222^^,^^228^^,^^232^^,^^247^^–^^249^^,^^257^	26
	2	Invest capacity in fostering change for different specializations and teams to create a trusting environment for knowledge-exchange, particularly around inefficiencies and pressure on grant funding ^23^^,^^26^^,^^41^^,^^43^^,^^45^^,^^49^^,^^59^^,^^87^^,^^89^^,^^98^^,^^99^^,^^101^^,^^109^^,^^164^^,^^177^^,^^220^^,^^222^^,^^223^^,^^226^^,^^235^^,^^236^^,^^243^^,^^258^	23
	3	Invest in leadership training and encourage a culture of knowledge sharing between senior leaders to foster a healthy work environment, in particular around Open Science, Open Research practice and competencies (including project management and oversight and training such as awareness and motivation) ^8^^,^^19^^,^^23^^,^^24^^,^^60^^,^^98^^,^^108^^,^^152^^,^^164^^,^^172^^,^^178^^,^^222^^,^^228^^,^^230^^,^^243^^,^^247^^–^^250^^,^^257^^,^^259^	21
	4	Implement an Inclusive leadership programme, and promote the benefits of a collaborative rather than competitive research culture ^19^^,^^57^^,^^59^^,^^60^^,^^107^^,^^130^^,^^154^^,^^158^^,^^172^^,^^225^^,^^227^^,^^229^^,^^230^^,^^241^^,^^259^	15
	5	Provide access to research capacity building activities that value research and provide access to resources ^19^^,^^90^^,^^164^^,^^212^^,^^217^^,^^223^^,^^241^^,^^243^^,^^258^	9
**Make use of or build on existing tools and initiatives to advance innovation**	1	Centralize computing and experimental infrastructure to engage core facilities to provide data services, including ways to enhance productivity through social media use (and digitalisation) ^3^^,^^6^^,^^8^^,^^21^^,^^23^^,^^24^^,^^26^^,^^50^^,^^84^^,^^87^^,^^88^^,^^91^^,^^92^^,^^97^^,^^98^^,^^109^^,^^139^^,^^154^^,^^227^^,^^233^^,^^236^^,^^245^^,^^246^^,^^250^^–^^252^^,^^254^^,^^255^	28
	2	Consider implementing a Complementary Capacity Building (CCB) programme to improve the sustainability of research partnerships (including productivity), with particular focus on LMIC research capacity (and identifying synergies between research and services for development (R&S4D) ^6^^,^^8^^,^^21^^,^^23^^,^^26^^,^^50^^,^^84^^,^^87^^,^^88^^,^^90^^–^^92^^,^^94^^,^^97^^,^^98^^,^^139^^,^^153^^,^^221^^,^^223^^,^^234^^,^^236^^,^^250^^–^^252^^,^^254^^,^^255^^,^^258^	27
	3	Promote or encourage use of: the Open Science Framework platforms; project management tools designed to enhance transparency and foster collaborations; the Open Innovation Science (OIS) concept/framework; Network data centres and task forces (e.g., UK Reproducibility Network and developing framework/guidelines to enhance understanding); and implement and encourage use of contributorship approaches such as mandating the use of CRediT ^7^^,^^24^^,^^107^^,^^130^^,^^178^^,^^215^^,^^233^^,^^260^	8
	4	Consider optimal models of collaboration which promote integration that is appropriate and relevant as different problems (including different countries) require different approaches ^154^^,^^221^^,^^223^^,^^225^^,^^228^^,^^233^	6

As the evidence has shown, working relationship challenges in research culture require structural changes by transforming people, places, and practices. ^247^ Any steps to reform will require accessibility to opportunities and resources that collectively bring research staff together in a unified and cohesive way to promote and create trust (rather than having intensive competitive pressures to achieve based on individual merit). ^51^^,^^164^^,^^247^

The evidence reveals multiple layers of complexity around the notion of ‘teamwork’ and the interrelated social and environmental factors that unfortunately reinforce a status quo. For change to occur there needs to be synergy for collaboration to ensure individuals, Research Performing Organisations (RPOs), Research Funding Organisations (RFOs) and society share a unified approach to move beyond solitary, isolated teams to a deeper integration of multi-disciplinary/inter-disciplinary culture. ^7^^,^^57^ An inclusive, representative, and collaborative research environment contributes to improvement in researchers’ sense of belonging and to positive cultural change. ^206^

The identified evidence suggests that there is a need to take a holistic and integrated view of the intrinsic (those within disciplines) and extrinsic factors (those outside of disciplines) that affect the research environment to come up with novel ways to tackle the challenges with teamwork and collaboration to ensure openness and a cultural shift in the right direction. ^7^ Given the growing evidence that success in research and innovation requires diversity in roles, knowledge, and skills, an inclusive, representative, and collaborative research environment contributes to improvement in researchers’ sense of belonging and to positive cultural change is required. ^206^

### Research quality and accountability – open and trustworthy research

From the existing evidence it was clear that transparency, open research, and integrity requires collaboration from RPOs, RFOs, researchers, publishers, and other sectoral organisations such as industry. ^27^^,^^260^^,^^261^ A large proportion of the evidence (more than a quarter of articles (133/253) included from the database searches and half of the grey literature (52/102)) highlighted several issues inhibiting open research practices, which some have termed as a ‘replication or reproducibility crisis’. ^24^^,^^86^^,^^216^^,^^235^^,^^237^^,^^257^^,^^262^ These increasing pressures on researchers suggest that it is causing a ‘publish or perish’ practice, and has meant that researchers are prioritising ‘getting it published’ rather than ‘getting it right’. ^24^^,^^86^^,^^235^^,^^237^^,^^254^^,^^255^^,^^257^

However, as noted by Munafo (2022), the replication crisis could be regarded as an opportunity to promote motivation for improvements. Determining where effort is most needed and what changes are required, not only provides opportunity for the research ecosystem but also how RPOs and RFOs can mandate open research practices, and therefore coordinate change at both research integrity and researcher integrity level ^261^ (see Table 5 for a summary of the key considerations associated to teamwork and supportive working relationships)

**Table 5.** Key concepts and statements associated to teamwork and supportive working relationships.

Key themes	No	Statements	No. refs
**Incentives and innovation**	1	Synthesize insights across multiple disciplines to help to unify collaborative practices and breakdown boundaries and disconnect to signal organisational values, such as the Open Innovation in Science (OIS) Research Framework (particularly for early career researchers, supervisors, technicians), enabling change to the research ecosystem becoming interoperable and responsive to the open assess movement ^7^^,^^8^^,^^19^^,^^23^^,^^26^^,^^43^^,^^49^^,^^55^^,^^84^^–^^86^^,^^92^^,^^98^^,^^101^^,^^109^^,^^119^^,^^126^^–^^128^^,^^151^^,^^156^^,^^192^^,^^224^^,^^234^^–^^236^^,^^241^^,^^263^^–^^278^	43
	2	Encourage greater efficiency and use of innovative and alternative approaches such as alternative publishing models (e.g., Octopus); registering with Center for Open Science; methods to assess research and researchers (e.g., SPACE); and, Open Knowledge Indicators, mapping diversity, communication and coordination ^23^^,^^55^^,^^85^^,^^108^^,^^127^^,^^151^^,^^162^^,^^169^^,^^208^^,^^216^^,^^237^^,^^247^^,^^256^^,^^261^^,^^263^^–^^265^^,^^279^^–^^291^	30
	3	Prioritise shared decision making to ensure all perspectives of the full research eco system are captured, to initiate change in practice, including policy makers, funders, publishers, technicians, researchers, institution leaders, editors, including level of appropriateness for performance based funding schemes ^5^^,^^26^^–^^28^^,^^36^^,^^84^^,^^85^^,^^92^^,^^98^^,^^127^^,^^138^^,^^151^^,^^156^^,^^224^^,^^233^^,^^236^^,^^244^^,^^257^^,^^268^^,^^278^^,^^283^^,^^287^^,^^288^^,^^292^^–^^296^	28
	4	Maintain hiring, appointment and promotional policies are fair and not solely based on authorship, publications or secured grants, and value softer skills ^24^^,^^55^^,^^85^^,^^135^^,^^136^^,^^169^^,^^216^^,^^231^^,^^237^^,^^247^^,^^256^^,^^257^^,^^263^^,^^264^^,^^275^^,^^279^^,^^297^^–^^306^	26
	5	Develop a coordinated approach to incentivize open access policies to optimise a positive cultural shift based on Government recognition of UK Research and Innovation’s position on open access research practice, (including European and international position and status of progression/advancement in open research) ^8^^,^^27^^,^^85^^,^^98^^,^^126^^,^^135^^,^^162^^,^^218^^,^^250^^,^^268^^,^^271^^,^^272^^,^^274^^,^^275^^,^^288^^,^^301^^,^^306^^,^^307^	18
	6	Ensure continued monitoring and evaluation, including meta-research/research on research takes place to avoid unintended consequences, efficient use of resources and demonstrate which aspects are beneficial to the research ecosystem (including where improvements are required at institutional and professional level) ^21^^,^^200^^,^^247^^,^^261^^,^^288^^,^^296^^,^^304^^,^^308^^–^^312^	12
**Creation and facilitation**	1	Monitor, evaluate and embed learning from education, training, supervision and mentoring to improve research integrity and to create a responsible research culture that is not individualized (including publishing culture built on individual reputation and rankings) but is a collective role in promoting and fostering research/academic integrity, through initiative such as open science peer networks, not to capitalize on individual researchers compliance ^4^^,^^19^^,^^21^^,^^26^^,^^43^^,^^55^^,^^76^^,^^85^^,^^98^^,^^101^^,^^106^^,^^108^^–^^110^^,^^119^^,^^121^^,^^126^^,^^127^^,^^129^^,^^138^^,^^151^^,^^169^^,^^185^^,^^210^^,^^234^^–^^236^^,^^241^^,^^247^^,^^250^^,^^253^^,^^256^^,^^257^^,^^261^^–^^264^^,^^268^^,^^272^^,^^277^^,^^278^^,^^280^^–^^283^^,^^285^^–^^287^^,^^289^^,^^294^^,^^300^^,^^308^^,^^309^^,^^311^^,^^313^^–^^331^	74
	2	Adopt open practices early on at all staff levels, but also at the institutional and funders level particularly around software and digital tools (including social media, Artificial Intelligence capabilities, the digital context, management tools), publishing mechanisms, workflows, ethics and data accessibility, supporting collaborations and training progression ^36^^,^^43^^,^^55^^,^^56^^,^^70^^,^^72^^,^^76^^,^^85^^,^^86^^,^^99^^,^^101^^,^^106^^,^^108^^,^^110^^,^^119^^,^^121^^,^^129^^,^^142^^,^^162^^,^^214^^,^^233^^,^^237^^,^^241^^,^^253^^,^^257^^,^^260^^,^^263^^,^^264^^,^^266^^,^^284^^,^^286^^,^^287^^,^^290^^–^^292^^,^^304^^,^^308^^,^^309^^,^^316^^,^^317^^,^^321^^,^^323^^,^^326^^,^^332^^–^^342^	71
	3	Ensure alignment between grant funding and publication outputs as well as consistency with open research initiatives, and opportunities to create mechanisms for reproducibility so greater collaboration can be gained, including understanding of authorship/contributorship consideration ^8^^,^^19^^,^^24^^,^^55^^,^^84^^,^^85^^,^^98^^,^^127^^,^^135^^,^^136^^,^^151^^,^^169^^,^^231^^,^^233^^,^^235^^,^^237^^,^^241^^,^^250^^,^^251^^,^^255^^–^^257^^,^^262^^,^^264^^,^^274^^,^^276^^,^^279^^,^^280^^,^^284^^–^^288^^,^^291^^,^^298^^,^^299^^,^^301^^,^^302^^,^^305^^,^^306^^,^^319^^,^^327^^,^^329^^,^^343^^,^^344^	46
	4	Coordinate and facilitate research integrity officers/champions to promote and create a responsible research culture, including opportunities for an academic integrity framework for policy and practice (including institutional improvements and avoiding the persistence of behaviors detrimental to reproducibility while encouraging responsible research conduct) ^19^^,^^21^^,^^28^^,^^43^^,^^76^^,^^84^^,^^98^^,^^101^^,^^106^^,^^110^^,^^119^^,^^121^^,^^127^^,^^151^^,^^210^^,^^235^^,^^236^^,^^251^^,^^255^^,^^262^^,^^272^^,^^277^^,^^278^^,^^280^^,^^285^^,^^286^^,^^288^^,^^294^^,^^296^^,^^309^^,^^311^^,^^313^^,^^314^^,^^318^^,^^322^^,^^324^^,^^326^^–^^331^^,^^345^	44
	5	Support Responsible Research Practices (RRP) as they require facilitation, advice and steer from the Government, funding organisations and academic institutions (progression and progress cannot be done in isolation). Such activity should consider six key areas: research policies; research practices; training researchers; evaluating research (ers); rewarding researchers; funding research (ers) ^4^^,^^27^^,^^43^^,^^56^^,^^70^^,^^72^^,^^76^^,^^85^^,^^86^^,^^92^^,^^98^^,^^99^^,^^101^^,^^106^^,^^108^^,^^110^^,^^119^^,^^121^^,^^129^^,^^156^^,^^185^^,^^233^^,^^247^^,^^277^^,^^281^^,^^286^^,^^294^^,^^298^^,^^307^^,^^309^^,^^314^^,^^315^^,^^323^^,^^325^^,^^326^^,^^346^	36
	6	Enable researchers to have a voice in articulating (and contextualizing) how research could be evaluated and provide a mechanism for more detailed and transparent reporting of scholarly activities, using formal evaluative systems that explicitly captures behaviors that support reproducibility ^36^^,^^84^^,^^127^^,^^128^^,^^131^^,^^151^^,^^227^^,^^235^^,^^251^^,^^255^^,^^262^^,^^272^^,^^273^^,^^276^^,^^280^^,^^285^^,^^286^^,^^288^^,^^297^^,^^312^^,^^327^^,^^329^^,^^332^^,^^333^^,^^347^^,^^348^	26
**Fostering transparency and visibility**	1	Provide clarity, transparency and understanding of research mandates, policies and procedures to permit and maintain productivity in research for all staff and students (including career advantages), across all disciplines (acknowledging the reproducibility networks) ^4^^,^^8^^,^^22^^,^^23^^,^^26^^,^^36^^,^^43^^,^^84^^,^^86^^,^^92^^,^^97^^,^^101^^,^^115^^,^^127^^,^^128^^,^^138^^,^^142^^,^^151^^,^^156^^,^^162^^,^^185^^,^^192^^,^^227^^,^^234^^–^^236^^,^^241^^,^^250^^–^^252^^,^^254^^,^^255^^,^^262^^,^^267^^,^^269^^,^^270^^,^^272^^–^^276^^,^^278^^,^^280^^,^^285^^,^^286^^,^^288^^,^^297^^,^^312^^,^^314^^–^^316^^,^^326^^,^^327^^,^^329^^,^^333^^,^^337^^,^^343^^,^^349^	58
	2	Support open research to allow research to be more reliable by sharing protocols, data, reproduction of analyses, and offers greater scrutiny to ensure good quality and replication of findings is critical to increase reliability and benefit all researchers, at all levels (making research accessible), including knowledge exchange ^8^^,^^19^^,^^36^^,^^84^^,^^85^^,^^98^^,^^127^^,^^128^^,^^142^^,^^151^^,^^162^^,^^208^^,^^233^^–^^235^^,^^250^^,^^251^^,^^253^^,^^255^^,^^257^^,^^262^^,^^273^^,^^276^^,^^280^^,^^281^^,^^285^^,^^286^^,^^288^^,^^290^^,^^297^^,^^298^^,^^301^^,^^304^^,^^314^^,^^316^^,^^317^^,^^321^^,^^327^^,^^329^^,^^332^^,^^333^^,^^335^^,^^337^^,^^339^^,^^341^^,^^343^^,^^347^^,^^350^^,^^351^	51
	3	Gain greater understanding and consideration of existing steps to promote open science practices such Center for Open Science and its pre-registration process ( https://cos.io/prereg/); Editor’s Code of Ethics ( http://editorethics.uncc.edu/); Committee of Publication Ethics (COPE, http://publicationethics.org/); Transparency and Openness Promotion (TOP) guidelines; Open Science Grid ( http://opensciencegrid.org/); Open Knowledge institutions (OKIs); European Network of Research Integrity; SPACE (SPACE is a rubric for analyzing institutional progress indicators and conditions for success); Open Government Data Act; FAIR (findable, accessible, interoperable, and reusable); alternative repositories for open access publications (University Journals) ^21^^,^^23^^,^^43^^,^^76^^,^^85^^,^^98^^,^^106^^,^^108^^,^^110^^,^^119^^,^^121^^,^^127^^,^^151^^,^^208^^,^^236^^,^^262^^,^^278^^,^^280^^–^^282^^,^^285^^–^^287^^,^^289^^,^^292^^,^^303^^,^^304^^,^^323^^,^^326^^–^^330^^,^^334^^,^^338^^,^^340^^,^^344^	38
	4	Increasingly adopt and promote publicly available data sets shared through repositories (e.g., Figshare, Zenodo), data management techniques, open materials and open data badges through the Center for Open Science, increasingly being mandated by funders and journals (including networks such as the Open Traits Network and toolkits for open access, and self-assessment of digital research) ^3^^,^^8^^,^^23^^,^^26^^,^^49^^,^^56^^,^^84^^,^^85^^,^^98^^,^^108^^,^^109^^,^^127^^,^^129^^,^^151^^,^^156^^,^^208^^,^^224^^,^^250^^,^^252^^,^^254^^,^^260^^,^^266^^,^^274^^,^^280^^,^^282^^,^^284^^–^^287^^,^^289^^,^^317^^,^^321^^,^^328^^,^^332^^,^^336^^,^^337^^,^^342^	37
	5	Ensure scholarly outputs are credited using alternative contributorship models (e.g., CREDiT) and moving away from the traditional authorship models including becoming more preventative than reactive ^21^^,^^24^^,^^76^^,^^85^^,^^136^^,^^231^^,^^279^^,^^284^^,^^288^^,^^298^^,^^302^^,^^305^	12
	6	Incorporate and consider web-based tools such as Open Science Framework (OSF), Open Knowledge Institutions framework (OKIs) to increase transparency and visibility of research at an international, global and institutional level ^23^^,^^108^^,^^127^^,^^208^^,^^260^^,^^280^^,^^282^^,^^285^^,^^286^^,^^289^^,^^317^^,^^343^	13
	7	Become a signatory of initiatives such as DORA involvement with the Reproducibility Networks at local, institutional and sectoral level (UKRN Committee, 2021) ^22^^,^^27^^,^^36^^,^^98^^,^^260^^,^^261^^,^^268^^,^^304^^,^^312^^,^^329^	10

The existing evidence demonstrated that open research practices (e.g., research integrity, researcher integrity, open data, open access and transparency) requires a global effort, as well as involvement from all sectors of the research ecosystem (e.g., institutions, researchers, funding organisations, publishers, industry). However, more evidence is needed to demonstrate where and in what circumstances the change is having tangible benefit. ^43^^,^^260^^,^^261^^,^^282^^,^^329^

As the evidence suggests, practices should be evaluated to assess whether change has been of value, enhancing the research pathway and algin to be evidence informed, therefore avoiding any unintended consequences. ^5^^,^^7^^,^^135^^,^^261^^,^^272^ Meta research (e.g., research on research, meta science) is one way to evaluate and evidence any innovation taking place, and therefore determine the impact and tangible benefit of these changes to promote and enhance the research ecosystem. ^7^^,^^24^^,^^36^^,^^247^^,^^261^^,^^281^^,^^329^^,^^341^

The evidence found several initiatives such as the UK Reproducibility Network (UKRN), Declaration on Research Assessment (DORA), and the European Network of Research Integrity (ENRI) to promote, encourage and prioritise the facilitation and creation of open research practices. ^7^^,^^22^^,^^281^^,^^285^^,^^325^ Adopting such initiatives enhances innovation across all aspects of the research ecosystem but there is variation on how far they have been implemented (including what stage of the development) and the acceptance level from researchers, RPOs and RFOs. ^303^^,^^322^^,^^325^^,^^330^

## Discussion

From the evidence, it was clear that there were several initiatives to seek a cultural change across the research institutions/Higher Education Institutions. Although this was promising to see, the commitment is complex considering the multifaceted structures and processes governing the research ecosystem. Adding to the complexity, is the acknowledgement from research institutions that they too have a role to play in not only supporting research staff, at all levels, but also recognise the role and function of research management staff. ^76^^,^^85^^,^^114^^,^^137^^,^^156^^,^^229^ As noted in the recent Research and Development report on people and culture strategy, high quality research and innovation requires an acknowledgment of the full range of people needed. ^8^ An inclusive, representative and collaborative research environment contributes to improvement in researchers’ sense of belonging and to positive cultural change. ^206^

The increasing competitiveness within the research environment, with research funding organisations (RFOs) placing greater focus on impact rather than creativity and innovation, is causing a global initiative for cultural change. The health of the research group and those that lead them has been identified as an area that universities need to pay more attention to, rather than centering on individual researchers, particularly in the context of preventing research misconduct. ^43^ Team leaders play an important role in creating trustful environments, which support knowledge exchange processes and open research ^164^ and crucially they act as mentors and role models for research integrity and working practices. ^43^^,^^92^

There was a strong sense and recognition of the value and importance of research capacity building, and the evidence clearly provided a wealth of initiatives to embark on. Interestingly, models, approaches and initiatives for capacity building have been reported by several countries. ^57^^,^^70^^,^^90^^,^^103^^,^^130^^,^^133^^,^^159^^,^^223^^,^^310^^,^^352^ Initiatives taking place in many countries, emphasising the benefits of learning from experiences in other countries was encouraging to report from the literature. Factoring some of these initiatives, it is evident that academia is confronting the challenges ‘head on’ to build a more sustainable and credible research environment. ^20^^,^^162^^,^^216^ Although this is promising, the evidence suggests that research institutions must not assume that ‘one size fits all’. There is diversity across disciplines, research staff (research, education, enterprise, and professional services) and several types of research institutions. To enhance research culture, different solutions will require different approaches at individual, institutional and systemic levels.

Over the last five years, and particularly the last two years (i.e., post the COVID-19 pandemic), there is a surge of evidence to capture the effects of these challenges and barriers and how these failings in our research ecosystem can be mitigated. Much of the literature focusing on career stability, job security and career progression suggested the need to build research capacity that spans across research, education, enterprise, and professional services. However, with this comes new challenges and pressures for teaching staff to also mentor, support and educate, whilst also having committed time to conduct their own research. ^7^^,^^24^^,^^44^^,^^50^^,^^73^^,^^74^^,^^97^^,^^177^^,^^194^^,^^337^

Guidance on how to create a global long-term sustainable model that has representation at all levels is going to take time, and the COVID-19 pandemic has aggravated this already challenging and highly pressured working environment. ^18^^,^^44^^,^^73^^,^^74^ The COVID 19 pandemic may have perhaps initiated more transparency on obtaining a work/life balance, particularly at a time when many parents across the world were not only managing increasing work demands but also having to manage home life and home schooling simultaneously. The effect of the COVID-19 pandemic has started to emerge in the literature, particularly how additional burden placed on women reduced their productivity far more than men with women having ‘borne the brunt of the pandemic in academic settings.’ ^177^ There has been a steady increase in mental health distress arising from COVID-19 pandemic reported, which added to the existing strain in academia can often feel detrimental to an individual’s career. ^18^

However, despite the pandemic causing global disruption and concern, it has initiated new opportunities to bring communities and countries together using digital tools. ^194^^,^^241^^,^^245^^,^^257^^,^^264^^,^^332^^,^^342^^,^^344^ Research, training and teaching took place online, and the evidence seemed to suggest that offering greater accessibility through virtual platforms goes some way to reform the connectivity and diversity of the research environment. A good example of this is virtual conferences, as those with accessibility issues, family commitments, funding limitations and research communities from Low- and Middle-Income Countries (LMIC) now can attend, where they could not before. ^208^^,^^245^ Any changes will inevitably take time, but small improvements over time can have an impact, affecting both research institutions and funding organisations. ^180^ In addition, the pandemic provided the impetus to embed kindness in research where empathy replaced the usual expectations on work-life balance, ^97^ and researchers who felt supported during the pandemic tended to have better indicators of well-being. ^73^^,^^74^

Given the growing evidence that success in research and innovation requires diversity in roles, knowledge, and skills, embedding a research culture of inequality between career types within the same research team discourages a culture of collaboration and appreciation of a diversity of roles, specialisations and contributions. ^41^^,^^164^ With increasing demands to incentivise and promote change it is necessary to acknowledge that both funding organisations and research institutions have responsibility to transform and shape best practice in research. Providing opportunities for research staff to combine their academic research with work in other sectors could bring more value to academia and strengthen the synergies for cultural change in the long-term. ^153^

### Study strengths and limitations

The main strength of the review was using several systematic database literature searches, which was complemented with additional grey literature searches of known online education articles and websites. However, a limitation is that scoping reviews only map the evidence and do not assess the quality of the articles or risk of bias. Most of the database articles had an international focus, with most of them from USA, Canada (28.0%) and other international countries (27.2%). For a large proportion of the grey literature, it was not possible to ascertain the articles countries region. Moreover, on a pragmatic basis, news items from news organisations based outside of UK, Europe, Norther America, and Australia were excluded, which along with the exclusion of non-English language documents means that other initiatives being used to improve research culture may well have been missed. On this basis, there could have been international and regional biases, but it could also be that there is a lack of evidence from these regions rather than missing articles from the systematic searches.

The review found more than 200 articles from the systematic database searches (n=253) and more than 100 from the grey literature searches (n=102) which suggested that there is growing literature around what constitutes a ‘good’ research culture and what this could look like. However, as the literature has shown, progress has been slow and although the evidence provided several examples of established initiatives and networking opportunities, the evidence was more anecdotal, and opinion focused.

## Conclusions

The review has shown that there is a wealth of evidence suggesting how and where changes are needed to establish a global cultural change to the research ecosystem. However, research organisations cannot act in isolation. Individuals, research organisation and funding organisations need to be responsible and work together; to uphold and ensure fair and transparent policies and governance. Change will not happen overnight, but by working together in a collaborative and diverse way to ensure all views, opinions and expectations are fully inclusive that strengthen and enhance research culture for the better.

The barriers to a sustainable research culture are complex and underneath linger more multi-faceted challenges, such as the impact on the wellbeing of research staff, resistance to innovation, equity for research institution staff and career progression. ^28^^,^^62^^,^^64^^,^^85^^,^^195^^,^^260^^,^^336^ Adding to the complexity is the increasing pressure for academic institutions, research groups, disciplines, and researchers to demonstrate impact. The growing focus on performance measures has undoubtedly caused unintended consequences for the whole research ecosystem. This model is not sustainable, not only for the quality of research and trust in research, but also for the next generation of talented researchers. Researchers are leaving academia, leaving behind a career that should be fostering innovation and building research capacity at its core. Removing such barriers and adopting best research practice and enhancing the diversity of opportunities for all is ultimately down to everyone working within the research environment.

## Ethics and consent

Ethical approval and written consent statement were not required.

## Authors’ contributions

**Conceptualization:** Amanda Blatch-Jones, Kay Lakin, Sarah Thomas

**Data curation:** Amanda Blatch-Jones, Kay Lakin

**Formal analysis:** Amanda Blatch-Jones, Kay Lakin

**Investigation:** Amanda Blatch-Jones, Kay Lakin

**Methodology:** Amanda Blatch-Jones, Kay Lakin

**Validation**: Amanda Blatch-Jones, Kay Lakin

**Project administration:** Amanda Blatch-Jones

**Supervision:** Amanda Blatch-Jones

**Writing – original draft:** Amanda Blatch-Jones

**Writing – review and editing:** Amanda Blatch-Jones, Kay Lakin, Sarah Thomas

## Data Availability

All underlying data are available as part of the article and no additional source data are required. OSF: A scoping review on what constitutes a good research culture. https://osf.io/wjhcf/. ^353^ This project contains the following underlying data:
• Fig 1 PRISMA flow diagram (PRISMA) • Fig 2 themed areas diagram (four areas of statements diagram) • S1 Appendix search terms and key words (search terms and key words) • S2 Appendix PRISMA ScR checklist (completed checklist) • S1 Table database searches examples (search strategies used) • S2 Table database lit articles (complete list of included articles in the review) • S3 Table grey lit articles (complete list of included articles from the grey literature in the review) Data are available under the terms of the Creative Commons Attribution 4.0 International license (CC-BY 4.0). ***Reporting guidelines*** OSF repository: PRISMA ScR checklist and flow chart for “A scoping review on what constitutes a good research culture.” https://osf.io/wjhcf/. ^353^ Data are available under the terms of the Creative Commons Attribution 4.0 International license (CC-BY 4.0).
