# Supplementary material for: A scoping review on what constitutes a good research culture
Source: F1000Res. 2024 Oct 14;13:324. Originally published 2024 Apr 23. [Version 3] doi: 10.12688/f1000research.147599.3 (PMC11140362; doi:10.12688/f1000research.147599.3)
Supplement: Peer review history

# Reviewer report: Reviewer response for version 3

**DOI:** 10.5256/f1000research.171769.r331577

**Published:** 2024-12-17

**Authors:** Stephen Curry

**Affiliations:** 1 Department of Life Sciences, Imperial College London, London, England, UK

My thanks to the authors for providing a revised version of their article in constructive response to my comments. I take their point that a scoping review is more of a survey than an analysis of practice and think that we are of one mind in hoping to see in future analyses the critical evaluation of policies and processes that aim to enhance our research cultures.

Are the rationale for, and objectives of, the Systematic Review clearly stated?

No

Is the statistical analysis and its interpretation appropriate?

Not applicable

If this is a Living Systematic Review, is the ‘living’ method appropriate and is the search schedule clearly defined and justified? (‘Living Systematic Review’ or a variation of this term should be included in the title.)

Not applicable

Are sufficient details of the methods and analysis provided to allow replication by others?

Yes

Are the conclusions drawn adequately supported by the results presented in the review?

Partly

Reviewer Expertise:

Research assessment; research on research.

I confirm that I have read this submission and believe that I have an appropriate level of expertise to confirm that it is of an acceptable scientific standard.

# Reviewer report: Reviewer response for version 3

**DOI:** 10.5256/f1000research.171769.r331579

**Published:** 2024-10-17

**Authors:** Elisabeth Grey

**Affiliations:** 1 Bristol Medical School and NIHR Applied Research Collaboration (ARC) West, University of Bristol, Bristol, England, UK

No further comments, the authors have addressed the reviewers comments.

Are the rationale for, and objectives of, the Systematic Review clearly stated?

Yes

Is the statistical analysis and its interpretation appropriate?

Not applicable

If this is a Living Systematic Review, is the ‘living’ method appropriate and is the search schedule clearly defined and justified? (‘Living Systematic Review’ or a variation of this term should be included in the title.)

Not applicable

Are sufficient details of the methods and analysis provided to allow replication by others?

Yes

Are the conclusions drawn adequately supported by the results presented in the review?

Yes

Reviewer Expertise:

Health behaviour, health communication, qualitative methods, systematic reviews

I confirm that I have read this submission and believe that I have an appropriate level of expertise to confirm that it is of an acceptable scientific standard.

# Reviewer report: Reviewer response for version 2

**DOI:** 10.5256/f1000research.168087.r300823

**Published:** 2024-07-24

**Authors:** Elizabeth Gadd

**Affiliations:** 1 Loughborough University, Loughborough, England, UK

Thanks to the authors for working hard to implement the recommendations of my review. I still couldn't see any references to the Vitae Research Culture Framework either in the references or the text which might be something worth revisiting given its significance in the research culture space.

Are the rationale for, and objectives of, the Systematic Review clearly stated?

Yes

Is the statistical analysis and its interpretation appropriate?

Not applicable

If this is a Living Systematic Review, is the ‘living’ method appropriate and is the search schedule clearly defined and justified? (‘Living Systematic Review’ or a variation of this term should be included in the title.)

Not applicable

Are sufficient details of the methods and analysis provided to allow replication by others?

Partly

Are the conclusions drawn adequately supported by the results presented in the review?

Yes

Reviewer Expertise:

Research assessment; research culture; scholarly communication; open research.

I confirm that I have read this submission and believe that I have an appropriate level of expertise to confirm that it is of an acceptable scientific standard.

# Response

**Published:** 2024-09-10

**Authors:** Amanda Blatch-Jones

**Affiliations:** Faculty of Medicine, University of Southampton, Southampton, Hampshire, UK

Comment : Thanks to the authors for working hard to implement the recommendations of my review. I still couldn't see any references to the Vitae Research Culture Framework either in the references or the text which might be something worth revisiting given its significance in the research culture space.

**Response : Thank you for picking this up. It has now been added to the introduction and referenced accordingly.**

# Reviewer report: Reviewer response for version 2

**DOI:** 10.5256/f1000research.168087.r300822

**Published:** 2024-07-16

**Authors:** Steven De Peuter

**Affiliations:** 1 KU Leuven, Leuven, Flanders, Belgium

Thank you for taking into account my comments and those of the other reviewers. I consider this version a substantial improvement from the previous. Allow me to formulate some additional minor comments that, when addressed, may further add to the paper’s clarity.

 There is some inconsistency between, on the one hand, the description of the *purpose *of the paper as formulated in the abstract and, on the other hand, the final paragraph of the Background section and the final sentence of the Methods section. Whereas the former places emphasis on poor research culture (“ *The purpose of the scoping review was to explore ‘What does the evidence say about the ‘problem’ with ‘poor’ research culture, what are the benefits of ‘good’ research culture, and what does ‘good’ look like?’”*), the Background and Method section focus more on *good* research culture. Furthermore, the text of the Results section primarily describes identified problematic aspects of the research culture, whereas the tables list considerations that will most likely improve the research culture and/or foster *good *culture. I definitely appreciate the positive approach of the considerations/recommendations so I wouldn’t change them. I think adding one or two sentences about where/how the different aspects of research culture are dealt with will assist the reader in taking in the extensive amount of information contained in the paper.

 Minor:

 BACKGROUND

 The subject and the verbs of the first sentence of the third paragraph of the Background section are not aligned:

 “consequences … does not only effect …, it” […consequences DO not only Affect, THEY … affect … reduce … affect; please also note the use of effect instead of affect]

 METHODS

*Eligibility criteria - participants*: The second sentence is odd, starting with “To be inclusive of…” followed by “to ensure”

 RESULTS

*Research quality and accountability*: “suggested a link between open research and reproducibility that could lead to public distrust” please specify how you see this link. I find it hard to imagine how openness would lead to public distrust, thinking it would make more sense to have a link between *lack of openness* and public distrust.

 Typos:

 Table 3

 theme 3 statement 2: space missing before opening bracket; better to use “reflecting” than “reflect”

 theme 3 statement 5: closing bracket missing

 table 4

 theme 1 first statement: “break down” instead of breakdown

 theme 1 statement 4: maintain … THAT are fair

 theme 2 statement 1: individual researchers’ compliance

 theme 2 statement 6: systems that explicitly capture (remove ‘s’) behaviors

 Theme 3 statement 3: …to promote open science practices such AS Center for Open Science

Are the rationale for, and objectives of, the Systematic Review clearly stated?

Yes

Is the statistical analysis and its interpretation appropriate?

Not applicable

If this is a Living Systematic Review, is the ‘living’ method appropriate and is the search schedule clearly defined and justified? (‘Living Systematic Review’ or a variation of this term should be included in the title.)

Not applicable

Are sufficient details of the methods and analysis provided to allow replication by others?

Yes

Are the conclusions drawn adequately supported by the results presented in the review?

Yes

Reviewer Expertise:

Research integrity, research culture, behavior change, health psychology, change management

I confirm that I have read this submission and believe that I have an appropriate level of expertise to confirm that it is of an acceptable scientific standard.

# Response

**Published:** 2024-09-10

**Authors:** Amanda Blatch-Jones

**Affiliations:** Faculty of Medicine, University of Southampton, Southampton, Hampshire, UK

**Reviewer's comment:** There is some inconsistency between, on the one hand, the description of the *purpose *of the paper as formulated in the abstract and, on the other hand, the final paragraph of the Background section and the final sentence of the Methods section. Whereas the former places emphasis on poor research culture (“ *The purpose of the scoping review was to explore ‘What does the evidence say about the ‘problem’ with ‘poor’ research culture, what are the benefits of ‘good’ research culture, and what does ‘good’ look like?’”*), the Background and Method section focus more on *good* research culture.

**Author response: **Thank you we have made all three areas (abstract, background and methods section the same) the same to be consistent about the focus of the review.

**Reviewer's comment: **Furthermore, the text of the Results section primarily describes identified problematic aspects of the research culture, whereas the tables list considerations that will most likely improve the research culture and/or foster *good *culture. I definitely appreciate the positive approach of the considerations/recommendations so I wouldn’t change them. I think adding one or two sentences about where/how the different aspects of research culture are dealt with will assist the reader in taking in the extensive amount of information contained in the paper.

**Author response: **Thank you for your comment. To put the considerations into context, the review scoped the ‘problems’ to determine probable considerations/recommendations, to understand what the benefits of a good research culture are and what it looks like (addressing the research questions).

**Reviewer's comment: **BACKGROUND

 The subject and the verbs of the first sentence of the third paragraph of the Background section are not aligned: “consequences … does not only effect …, it” […consequences DO not only Affect, THEY … affect … reduce … affect; please also note the use of effect instead of affect]

 METHODS

*Eligibility criteria - participants*: The second sentence is odd, starting with “To be inclusive of…” followed by “to ensure”

 RESULTS

*Research quality and accountability*: “suggested a link between open research and reproducibility that could lead to public distrust” please specify how you see this link. I find it hard to imagine how openness would lead to public distrust, thinking it would make more sense to have a link between *lack of openness* and public distrust.

**Author response: **Thank you for drawing our attention to these three areas within the article. We have adjusted the sentence/s accordingly based on your suggestions. 

 Thank you for highlighting the typos and we have now corrected them.

# Reviewer report: Reviewer response for version 1

**DOI:** 10.5256/f1000research.161809.r271033

**Published:** 2024-07-04

**Authors:** Stephen Curry

**Affiliations:** 1 Department of Life Sciences, Imperial College London, London, England, UK

This is a review of the peer-reviewed and grey literature on research culture issues at research institutions that aims to explore the evidence base for (i) claims of poor research culture, (ii) the benefits of a ‘good’ research culture, and (iii) what ‘good’ might might look like.

 The review is systematic in its consideration of the literature published between 2017 and 2022 and certainly addresses an important and topical area. However, I have a number of concerns about the design and execution of the study.

 First, the aims lack focus. As stated, they are to “explore the interplay between research culture, open research, career paths, recognition and rewards and EDI”. This maps out an extremely broad range of issues and doesn’t clearly articulate the desired endpoints.

 A second concern is the fact that the literature surveyed is confined to a 5-year period that ended over two years ago. Given the topicality of the subject, I find this a strange limitation. What reassurance can the authors offer that their review is not already out of date?

 Third, and perhaps most critically, the report is largely descriptive and lacks an analytical edge that I think would add considerably to the value and impact of the piece. I was left wondering who is going to read this and what will they take away? The main conclusion in the abstract is that research institutions and funders need to work together, a finding that seems obvious given the systemic nature of the issues under discussion. What is new here that we didn’t know before the review was conducted?

 I suspect the lack of analysis may reflect the fact that the interactions between open research, EDI etc and research culture are extremely difficult to isolate and distinguish and that there have been relatively few efforts to evaluate whether interventions have had positive or negative effects on research culture. The problem is made more difficult because of the existence of external influences (e.g. funder policies, league tables, international competition) on institutional culture. Nevertheless, even to highlight the absence of such evaluation efforts would be a valuable point to make – and I hope that the authors might address this question in a revised version of the paper.  

 I have a few additional comments:

 In the first paragraph of the introduction, I think it would be more appropriate to present the ‘actions’ that have been taken in chronological order.

 Later in the introduction, it is stated that “attempts on reform requires commitment from everyone”. While I agree with this claim, it sits oddly with the study design, which is focused only on research institutions. This is one of several instances where I lost sight of the coherence of the arguments being presented.

 In the first paragraph of the section on Security and Career Progression, the authors identify “concerns over job security, career progression and sustainability…” but it is not clear to me what is meant by sustainability in this context.

 Later in this section it is stated that “The evidence suggests that offering potential solutions or supportive actions for academic institutions and the research community may enhance and stabilise career paths, particularly those in the early career stage, including those in technical and managerial roles.” This is a rather vague and weak statement. What solutions have been identified? Who is responsible for them and how effective are they? What does it mean to enhance a career path?

 On page 19 it is stated (with regard to the replication crisis) that “Determining where effort is most needed and what changes are required, not only provides opportunity for the research ecosystem but also how RPOs and RFOs can mandate open research practices, and therefore coordinate change at both research integrity and researcher integrity level…”. This is another rather vague claim where analysis is lacking. What specific changes might be made? Could the authors also address the very live debates around the merits and demerits of using mandates?

 In the Discussion, the sentence “The increasing competitiveness within the research environment, with research funding organisations (RFOs) placing greater focus on impact rather than creativity and innovation, is causing a global initiative for cultural change” doesn’t make sense to me. Is it not increasing awareness of the harmful impacts of research competition that is leading many stakeholders across the world to focus attention on improving research culture. This is just one example of what I find to be a rather fuzzy style of writing. This sense is compounded by the repetition of the phrase “the evidence suggests”. As noted above, I would like to see the authors doing a better job of sifting and weighing the evidence.

 The last paragraph of the Conclusions is not really a conclusion. It is to my mind a description of a situation that was evident before reading this review. The first paragraph begins with the statement that “The review has shown that there is a wealth of evidence suggesting how and where changes are needed to establish a global cultural change to the research ecosystem. ” I think it would add a great deal of value to the review if the authors could identify what they think are the most important changes. This would have the potential to focus the attention of the sector on the most urgent actions to be undertaken.

Are the rationale for, and objectives of, the Systematic Review clearly stated?

No

Is the statistical analysis and its interpretation appropriate?

Not applicable

If this is a Living Systematic Review, is the ‘living’ method appropriate and is the search schedule clearly defined and justified? (‘Living Systematic Review’ or a variation of this term should be included in the title.)

Not applicable

Are sufficient details of the methods and analysis provided to allow replication by others?

Yes

Are the conclusions drawn adequately supported by the results presented in the review?

Partly

Reviewer Expertise:

Research assessment, EDI, metascience, structural biology

I confirm that I have read this submission and believe that I have an appropriate level of expertise to confirm that it is of an acceptable scientific standard, however I have significant reservations, as outlined above.

# Response

**Published:** 2024-08-19

**Authors:** Amanda Blatch-Jones

**Affiliations:** Faculty of Medicine, University of Southampton, Southampton, Hampshire, UK

Unfortunately we did not receive your review until we submitted a revised version. We will of course address your comments in version 3, when we have received all of the reviewers comments. Thank you again for your comments and suggestions for improvements.

# Response

**Published:** 2024-09-10

**Authors:** Amanda Blatch-Jones

**Affiliations:** Faculty of Medicine, University of Southampton, Southampton, Hampshire, UK

In addition to our previous comments we would like to add that we have addressed your comments in line with the other comments and feedback received. Thank you for your comments. 

 First, the aims lack focus. As stated, they are to “explore the interplay between research culture, open research, career paths, recognition and rewards and EDI”. This maps out an extremely broad range of issues and doesn’t clearly articulate the desired endpoints.

**Thank you for your comment. The scoping review was kept broad for the purposes of the review and the related areas highlighted in the Wellcome report. On this basis, we kept the review broad to capture relevant evidence (and contribute to the wider piece of work the review contributed to).**

 A second concern is the fact that the literature surveyed is confined to a 5-year period that ended over two years ago. Given the topicality of the subject, I find this a strange limitation. What reassurance can the authors offer that their review is not already out of date?

**We appreciate your concern around the period; however, this was chosen to consider existing literature in the space, the Wellcome report and initiatives in research culture. The period covered relevant references and included reports of changes during COVID-19 pandemic. As this was a scoping review to determine the existing evidence, we were only covering the specified areas within that period.**

 Third, and perhaps most critically, the report is largely descriptive and lacks an analytical edge that I think would add considerably to the value and impact of the piece. I was left wondering who is going to read this and what will they take away? The main conclusion in the abstract is that research institutions and funders need to work together, a finding that seems obvious given the systemic nature of the issues under discussion. What is new here that we didn’t know before the review was conducted?

**The review provides updated literature from the Wellcome report and supports the existing evidence that although there are further developments in this space, there is still a lack of collaborative working. The statements provided in the tables gives greater clarity around what these developments are and how they could be considered to foster and incentivise what a good research culture looks like. As this was a scoping review, analytical content or assessment was out of scope.**

 I suspect the lack of analysis may reflect the fact that the interactions between open research, EDI etc and research culture are extremely difficult to isolate and distinguish and that there have been relatively few efforts to evaluate whether interventions have had positive or negative effects on research culture. The problem is made more difficult because of the existence of external influences (e.g. funder policies, league tables, international competition) on institutional culture. Nevertheless, even to highlight the absence of such evaluation efforts would be a valuable point to make – and I hope that the authors might address this question in a revised version of the paper.  

**As noted in the review, research culture is in itself complex due to multiple factors at play. The review highlights these complexities and the range of areas required to promote and encourage a good research culture. We agree with your comment and have highlighted these concerns within the revised version, thank you.**

 In the first paragraph of the introduction, I think it would be more appropriate to present the ‘actions’ that have been taken in chronological order.

 Later in the introduction, it is stated that “attempts on reform requires commitment from everyone”. While I agree with this claim, it sits oddly with the study design, which is focused only on research institutions. This is one of several instances where I lost sight of the coherence of the arguments being presented.

**Thank you for your comment. Addressing this point in the introduction is a valid point, demonstrating how the Wellcome report highlighted the need for greater commitment from not just research institutions (and demonstrating the complexity).**

 In the first paragraph of the section on Security and Career Progression, the authors identify “concerns over job security, career progression and sustainability…” but it is not clear to me what is meant by sustainability in this context.

**Thank you. We have added some examples of what we mean by sustainability in this context.**

 Later in this section it is stated that “The evidence suggests that offering potential solutions or supportive actions for academic institutions and the research community may enhance and stabilise career paths, particularly those in the early career stage, including those in technical and managerial roles.” This is a rather vague and weak statement. What solutions have been identified? Who is responsible for them and how effective are they? What does it mean to enhance a career path?

**We have referred the reader to the table, which provides the evidence for this section. We didn’t elaborate within the text as we would be duplicating what is reported in table 2. Several solutions are reported in the table, and as reported, a range of individuals are responsible for initiating them (individuals themselves, research organisations, departments, HR etc).**

 On page 19 it is stated (with regard to the replication crisis) that “Determining where effort is most needed and what changes are required, not only provides opportunity for the research ecosystem but also how RPOs and RFOs can mandate open research practices, and therefore coordinate change at both research integrity and researcher integrity level…”. This is another rather vague claim where analysis is lacking. What specific changes might be made? Could the authors also address the very live debates around the merits and demerits of using mandates?

**Thank you for your comment. Like the above statement, the scoping review provided a summary of the evidence found rather than analysing the evidence (another type of review approach would be needed for this). The purpose here was to highlight and assess the scope of the literature and map it to specific areas (the four areas reported in the Wellcome report).**

 In the Discussion, the sentence “The increasing competitiveness within the research environment, with research funding organisations (RFOs) placing greater focus on impact rather than creativity and innovation, is causing a global initiative for cultural change” doesn’t make sense to me. Is it not increasing awareness of the harmful impacts of research competition that is leading many stakeholders across the world to focus attention on improving research culture. This is just one example of what I find to be a rather fuzzy style of writing. This sense is compounded by the repetition of the phrase “the evidence suggests”. As noted above, I would like to see the authors doing a better job of sifting and weighing the evidence.

**This section has been revised in a previous version. However, weighing or providing an analytical account of the included evidence is out of scope for scoping reviews. Scoping reviews aim to understand *‘What has been done previously?’* and *‘What does the literature say?’* compared to systematic reviews that ask the question *‘Does this intervention work for this group of individuals?***

 The last paragraph of the Conclusions is not really a conclusion. It is to my mind a description of a situation that was evident before reading this review. The first paragraph begins with the statement that “The review has shown that there is a wealth of evidence suggesting how and where changes are needed to establish a global cultural change to the research ecosystem. ” I think it would add a great deal of value to the review if the authors could identify what they think are the most important changes. This would have the potential to focus the attention of the sector on the most urgent actions to be undertaken.

**Thank you for your comment. We have extended the conclusions and included the Vitae citation.**

**However, we have not directed the reader to specific priorities as we feel this would add bias to the review. As already noted there are several areas in the tables under the four areas that would be considered to some as priority but may not be to someone else. On this basis, it would be the readers decision to understand what is priority to them and their institution based on what is feasible and within their control.**

# Reviewer report: Reviewer response for version 1

**DOI:** 10.5256/f1000research.161809.r277505

**Published:** 2024-05-30

**Authors:** Elizabeth Gadd

**Affiliations:** 1 Loughborough University, Loughborough, England, UK

Thank you for the opportunity to review this scoping review on what constitutes a good research culture, and thank you to the authors for taking the time to share their review with a wider audience through this piece.

 I make a number of minor comments against some of the article's headings below, however I think there are some more fundamental issues that it would be helpful to address to secure the scientific soundness of this article and therefore its contribution to the literature. 

**1) Clarity & consistency around the dimensions of research culture being investigated**

 It's not clear how the particular lenses of open research, career research paths (recognition & awards) & EDI mentioned in the background section were chosen given the research question appears to put no boundaries around the elements of poor research culture & the benefits of a good culture and what it looks like. In the methods section, the dimensions of research culture under investigation appear to have changed to 'four key areas highlighted from existing published work from the Wellcome Trust'. The argument given is that it will ensure consistency and continuity to predefined areas already established by the research environment.' (I'm not sure to what the authors refer when they use the phrase 'the research environment' here.) It's unclear at this point what these four areas are, and why they were selected. Again, why be guided by the WT if the research question is to consider the problems and benefits of poor/good research cultures more broadly?  It would be helpful if a stronger case was made for the dimensions of research culture being used to analyse the literature given the specific research questions of the study.

**2) The use of the research questions as a study frame**

 Having stated the research questions, they don't seem to provide a strong framework for the rest of the article. For example, Figure 2 summarises the evidence relating to 'what makes a good research culture' but not relating to the other elements of the research question (what are the problems with research culture and what benefits does it bring). If all three questions were being considered together, the heading of the figure could be changed.

 The research questions are not referred back to in the findings, discussion or conclusions, which leads to a loss of structure and clarity. 

**3) Superficial and incomplete analyses of the literature reviewed**

 Some of the evidence summaries feel superficial and possibly incomplete. Take as an example the sentence: "Evidence suggests that this [evaluating academic performance based on the use of inadequate proxies] can result in a lack of workload oversight, a culture discouraging of appreciation, that in turn makes researchers feel pressured to be successful, often resulting in a significant amount of time in pursuit of success at the cost of their wellbeing."  I know this literature quite well, and I don't think a lack of workload oversight and a culture of discouraging appreciation are the key issues with the overuse of publication metrics. 

 Again, in the Wellbeing & equality of opportunity section the authors write: 'Researchers are incentivized to attain research excellence, which can often result in hyper-competitiveness and unfair working practices.' However, it's generally agreed that the problem lies not so much in the pursuit of excellence so much as how narrowly excellence is defined.

 I provide some other examples below but this is not a complete list. 

**4) Poor phraseology**

 The findings of the review are often obscured by somewhat tortuous phrasing and unclear writing. For example, in the research quality and accountability statements table, one statement reads: 'Develop a coordinated approach to incentivize open access policies to optimise a positive cultural shift based on Government recognition of UK Research and Innovation’s position on open access research practice, (including European and international position and status of progression/advancement in open research)'. It's not clear to me what this is saying, nor whether reference to one nation's policy position is meaningful.

 Again, in the Wellbeing & equality of opportunity section, one sentence reads: 'As the evidence has shown, working relationship challenges in research culture require structural changes by transforming people, places, and practices.'

 I provide some other examples below, but this is by no means a complete list.

 I think it would be helpful if the authors scrutinise every sentence to ask, is this strictly accurate? How might this be misinterpreted? Am I representing the authors of the literature carefully? Have we identified the major themes? 

**5) Stronger discussion & conclusions**

 Finally, I find the discussion section rather unstructured and unclear. New points are introduced in the discussion that are not reported in the findings, e.g., reference to digital tools. The reader is not left with a clear sense as to what the authors proposed answers to the research questions are. 

**Minor corrections**

**Background**

 Minor Correction: "Funding organisations such as UK Research and Innovation (UKRI) enhanced its 2021-2022 allocation of research culture funding to Higher Education Providers (HEPs) to further explore research processes and experiences of working in research, through piloting new initiatives or enhancing existing activities."  - this funding is from Research England to English HEPs:

 Minor: "The consequences and challenges associated to an inadequate research culture is well evidence" - should be 'associated with' and 'are well evidenced'

**Methods**

 Minor: The term 'non-academic staff' is largely seen as derogatory - defining a group of people by what they are not. Research-enabling staff is a term in more common parlance now. Or professional services if you are looking more broadly.

**Search strategy**

 Minor: citations should be references I think?

**Data extraction & evidence selection**

 Minor: Citation needed for Rayyan

 Minor: I appreciate the work was carried out prior to the Vitae Research Culture study which identified 4 different core domains, however it would be good to mention this work as there is a lot of overlap with this paper.

- How research is managed and undertaken
- How research ensures value
- How people are supported
- How individuals engage with others

Minor: the numbers in the PRISMA flow diagram don't add up. The authors should take another look at this.

**Characteristics of the included studies**

 Minor: It should read 'conference papers' not 'conference proceedings'.

 Minor: UK is part of Europe. Is the 17 a subset of the 36 or separate to?

**Summarising the evidence**

 Minor: Could be clearer as to what you mean by 'career type'. Career pathway? Job family?

 Minor: I'm not sure what the term 'statements' refers to in Table 2. 'Statement' implies a citation from the literature, but these are very complex so I don't think that's what's being provided. 

**Wellbeing and equality of opportunity**

 Minor: just be careful with phrasing - 'The pandemic also initiated ‘kindness in research’' - I think you mean that the pandemic led to calls for more kindness in research? When writing reviews of this type, it's important to be strictly accurate as to exactly what was being suggested.

 Minor: Para beginning 'These practices' - it's not clear to what practices this refers?

**Research quality & accountability**

 Minor: This isn't quite accurate. 'the evidence...highlighted several issues inhibiting open research practices, which some have termed as a ‘replication or reproducibility crisis’. I think you mean that a link was made between open research and reproducibiity, but not that open research practices were termed a 'replication or reproducibilty crisis'? 

**Discussion**

 Minor: The report mentioned here: 'As noted in the recent Research and Development report on people and culture strategy, high quality research and innovation requires an acknowledgment of the full range of people needed. ^8'^

 is not a Research & Development report, but the BEIS R&D People & Culture Strategy.

 Minor: To what research group does this refer? 'The health of the research group and those that lead them has been identified as an area that universities need to pay more attention to'.

 Minor: The term 'impact' should be clarified throughout the article. In REF the term is used to describe non-academic impact. However, in the following sentence I think it refers to citation impact? ''The increasing competitiveness within the research environment, with research funding organisations (RFOs) placing greater focus on impact rather than creativity and innovation, is causing a global initiative for cultural change.' Either way, I'm not sure this sentence is strictly accurate.

Are the rationale for, and objectives of, the Systematic Review clearly stated?

Yes

Is the statistical analysis and its interpretation appropriate?

Not applicable

If this is a Living Systematic Review, is the ‘living’ method appropriate and is the search schedule clearly defined and justified? (‘Living Systematic Review’ or a variation of this term should be included in the title.)

Not applicable

Are sufficient details of the methods and analysis provided to allow replication by others?

Partly

Are the conclusions drawn adequately supported by the results presented in the review?

Yes

Reviewer Expertise:

Research assessment; research culture; scholarly communication; open research.

I confirm that I have read this submission and believe that I have an appropriate level of expertise to confirm that it is of an acceptable scientific standard, however I have significant reservations, as outlined above.

# Response

**Published:** 2024-06-18

**Authors:** Amanda Blatch-Jones

**Affiliations:** Faculty of Medicine, University of Southampton, Southampton, Hampshire, UK

**Reviewer comment: **1) ****Clarity & consistency around the dimensions of research culture being investigated

 It's not clear how the particular lenses of open research, career research paths (recognition & awards) & EDI mentioned in the background section were chosen given the research question appears to put no boundaries around the elements of poor research culture & the benefits of a good culture and what it looks like.

**Author response: **These areas were chosen as a result of the Wellcome Trust survey and the areas reported in their report. The review focused on what the solutions could be rather than focusing on the barriers and challenges to a good research culture (due to the existing coverage in the literature). The aim of the review was to therefore consider the evidence about solutions and what the research community could consider. However, we have amended the last paragraph to add some additional context to address your comment.

**Reviewer comment: **In the methods section, the dimensions of research culture under investigation appear to have changed to 'four key areas highlighted from existing published work from the Wellcome Trust'. The argument given is that it will ensure consistency and continuity to predefined areas already established by the research environment.' (I'm not sure to what the authors refer when they use the phrase 'the research environment' here.) It's unclear at this point what these four areas are, and why they were selected. Again, why be guided by the WT if the research question is to consider the problems and benefits of poor/good research cultures more broadly?  It would be helpful if a stronger case was made for the dimensions of research culture being used to analyze the literature given the specific research questions of the study.

**Author response: **We have revised the manuscript to be more descriptive of what the four areas are, along with what we mean by research environment (changed to research community based on the participants and respondents to the Wellcome Trust research cultures work).

**Reviewer comment:** 2) The use of the research questions as a study frame

 Having stated the research questions, they don't seem to provide a strong framework for the rest of the article. For example, Figure 2 summarises the evidence relating to 'what makes a good research culture' but not relating to the other elements of the research question (what are the problems with research culture and what benefits does it bring). If all three questions were being considered together, the heading of the figure could be changed.

**Author response: **The challenges and problems around research culture are well documented in the literature and we approached the research questions simultaneously, with more focus on solutions and what good research culture could look like. For each of the four areas, we have provided a summary of the challenges to address this part of the research question, with the main emphasis being the second part of the research question. The tables and figure 2 purposively focus on what constitutes a good research culture to address the challenges and barriers, to encourage a cultural shift with actionable actions. The purpose of the review was to focus on solutions rather than what the current challenges are, as these are well documented.

**Reviewer comment:** The research questions are not referred back to in the findings, discussion or conclusions, which leads to a loss of structure and clarity. 

**Author response: **We have made this clearer in the revised manuscript and referred back to the research questions.

**Reviewer comment:** 3) Superficial and incomplete analyses of the literature reviewed

 Some of the evidence summaries feel superficial and possibly incomplete. Take as an example the sentence: "Evidence suggests that this [evaluating academic performance based on the use of inadequate proxies] can result in a lack of workload oversight, a culture discouraging of appreciation, that in turn makes researchers feel pressured to be successful, often resulting in a significant amount of time in pursuit of success at the cost of their wellbeing."  I know this literature quite well, and I don't think a lack of workload oversight and a culture of discouraging appreciation are the key issues with the overuse of publication metrics. 

 Again, in the Wellbeing & equality of opportunity section the authors write: 'Researchers are incentivized to attain research excellence, which can often result in hyper-competitiveness and unfair working practices.' However, it's generally agreed that the problem lies not so much in the pursuit of excellence so much as how narrowly excellence is defined.

 I provide some other examples below but this is not a complete list. 

**Author response: **Thank you for your comments and feedback. We have revised the two areas you mentioned and have carefully considered and reviewed the rest of the manuscript.

**Reviewer comment: **4) Poor phraseology

 The findings of the review are often obscured by somewhat tortuous phrasing and unclear writing. For example, in the research quality and accountability statements table, one statement reads: 'Develop a coordinated approach to incentivize open access policies to optimise a positive cultural shift based on Government recognition of UK Research and Innovation’s position on open access research practice, (including European and international position and status of progression/advancement in open research)'. It's not clear to me what this is saying, nor whether reference to one nation's policy position is meaningful.

**Author response: **Thank you. We have amended the statement to make it clearer to the reader.

**Reviewer comment: **Again, in the Wellbeing & equality of opportunity section, one sentence reads: 'As the evidence has shown, working relationship challenges in research culture require structural changes by transforming people, places, and practices.'

**Author response: **We have amended the sentence to be more cohesive.

**Reviewer comment: **I provide some other examples below, but this is by no means a complete list.

 I think it would be helpful if the authors scrutinize every sentence to ask, is this strictly accurate? How might this be misinterpreted? Am I representing the authors of the literature carefully? Have we identified the major themes? 

**Author response: **Thank you. We have reviewed the manuscript and made several changes to make it more readable and coherent.

**Reviewer comment:** 5) Stronger discussion & conclusions

 Finally, I find the discussion section rather unstructured and unclear. New points are introduced in the discussion that are not reported in the findings, e.g., reference to digital tools. The reader is not left with a clear sense as to what the authors proposed answers to the research questions are. 

**Author response: **We have revised the discussion and conclusion to make it clearer to the reader and follow a more structured approach.

**Reviewer comment: Minor corrections**

**Background**

 Minor Correction: "Funding organizations such as UK Research and Innovation (UKRI) enhanced its 2021-2022 allocation of research culture funding to Higher Education Providers (HEPs) to further explore research processes and experiences of working in research, through piloting new initiatives or enhancing existing activities."  - this funding is from Research England to English HEPs:

**Author response: **Thank you. We have addressed this correction and amended the sentence accordingly.

**Reviewer comment: **Minor: "The consequences and challenges associated to an inadequate research culture is well evidence" - should be 'associated with' and 'are well evidenced'

**Author response: **Thank you, we have amended accordingly.

**Reviewer comment: Methods**

 Minor: The term 'non-academic staff' is largely seen as derogatory - defining a group of people by what they are not. Research-enabling staff is a term in more common parlance now. Or professional services if you are looking more broadly.

**Author response: **Thank you. We have replaced with research-enabling staff due to the scope of the review.

**Reviewer comment: **Search strategy

 Minor: citations should be references I think?

**Author response: **We have amended the manuscript to refer to references rather than citations to make it clearer to the reader.

**Reviewer comment: **Data extraction & evidence selection

 Minor: Citation needed for Rayyan

**Author response:** A link to Rayyan is now included.

**Reviewer comment: **Minor: I appreciate the work was carried out prior to the Vitae Research Culture study which identified 4 different core domains, however it would be good to mention this work as there is a lot of overlap with this paper.

- How research is managed and undertaken
- How research ensures value
- How people are supported
- How individuals engage with others

**Author response: **This has now been included in the manuscript as part of the overall introduction and discussion.

**Reviewer comment: **Minor: the numbers in the PRISMA flow diagram don't add up. The authors should take another look at this.

**Author response: **Thank you for noticing this. We have rectified the error in the PRISMA flow diagram and have updated accordingly.

**Reviewer comment: Characteristics of the included studies**

 Minor: It should read 'conference papers' not 'conference proceedings'.

**Author response: **We have amended to reflect your comment.

**Reviewer comment: **Minor: UK is part of Europe. Is the 17 a subset of the 36 or separate to?

**Author response: **We separated UK from Europe so that we could see what initiatives were specifically being conducted in the UK as funding, academic institutions etc work differently across country. As reported the 17 UK are separate to Europe, so the total across Europe was 43). We have adjusted the manuscript to be more concise.

**Reviewer comment: **Summarizing the evidence

 Minor: Could be clearer as to what you mean by 'career type'. Career pathway? Job family?

**Author response: **We have provided an explanation with examples on the first mention of career type.

**Reviewer comment: **Minor: I'm not sure what the term 'statements' refers to in Table 2. 'Statement' implies a citation from the literature, but these are very complex so I don't think that's what's being provided. 

**Author response: **We have changed ‘statements’ for all tables and have amended with key areas and considerations associated to [what the theme was].  

**Reviewer comment: **Wellbeing and equality of opportunity

 Minor: just be careful with phrasing - 'The pandemic also initiated ‘kindness in research’' - I think you mean that the pandemic led to calls for more kindness in research? When writing reviews of this type, it's important to be strictly accurate as to exactly what was being suggested.

 Minor: Para beginning 'These practices' - it's not clear to what practices this refers?

**Author response: **Thank you. We have amended the sentences accordingly.

**Reviewer comment:** Research quality & accountability

 Minor: This isn't quite accurate. 'the evidence...highlighted several issues inhibiting open research practices, which some have termed as a ‘replication or reproducibility crisis’. I think you mean that a link was made between open research and reproducibility, but not that open research practices were termed a 'replication or reproducibility crisis'? 

**Author response: **Thank you. We have amended the sentence accordingly.

**Reviewer comment: **Discussion

 Minor: The report mentioned here: 'As noted in the recent Research and Development report on people and culture strategy, high quality research and innovation requires an acknowledgment of the full range of people needed. ^8^ ^'  ^is not a Research & Development report, but the BEIS R&D People & Culture Strategy.

**Author response: **We have amended the manuscript accordingly.

**Reviewer comment: **Minor: To what research group does this refer? 'The health of the research group and those that lead them has been identified as an area that universities need to pay more attention to'.

**Author response: **We have provided more details to make it clearer to the reader.

**Reviewer comment: **Minor: The term 'impact' should be clarified throughout the article. In REF the term is used to describe non-academic impact. However, in the following sentence I think it refers to citation impact? ''The increasing competitiveness within the research environment, with research funding organizations (RFOs) placing greater focus on impact rather than creativity and innovation, is causing a global initiative for cultural change.' Either way, I'm not sure this sentence is strictly accurate.

**Author response: **We have adjusted and amended the manuscript where necessary.

# Reviewer report: Reviewer response for version 1

**DOI:** 10.5256/f1000research.161809.r277499

**Published:** 2024-05-28

**Authors:** Elisabeth Grey

**Affiliations:** 1 Bristol Medical School and NIHR Applied Research Collaboration (ARC) West, University of Bristol, Bristol, England, UK

Thank you for asking me to review this interesting scoping review on research culture in research institutions. The manuscript is well-written and comprehensive, providing clear details on the methods and supplementary materials to enable other researchers to replicate the searches. I only have a few very minor suggestions that I think would help clarify a few things in the text:

 In the Abstract, there are two stated aims, one under Background and one under Aims – these are not quite the same and I think it would be better to stick with one or the other (the first matches what is in the main text).

 Inclusion criteria or Types of sources – I think this needs a statement on the types of initiative that were studied e.g., ‘all initiatives aimed, partly or wholly, at improving research culture were included’. Or this may need to broader if reports were included about initiatives that did not specifically aim to improve research culture, but the impact on this was measured.

 In the PRISMA flow diagram, the reasons for exclusion are only provided for the academic records – could they be added for the grey records too?

 You mention that many of the articles were relevant to more than one of the four focus areas – could you add a little to the Methods to explain how you decided which area to categorise a report under in these cases? Or were they included in the synthesis for each category they related to (would be good to state this)?

 There is a typo in the fourth paragraph under ‘Research quality and accountability’ – ‘algin’?

 Table 4 and Table 5 have the same title – I think Table 5 should be Key concepts and statements associated with research quality and accountability

 It struck me that none of the concepts under ‘Security and career progression’ seemed to relate to job security, only career progression. This would be worth highlighting – ultimately to improve job security, research institutions and funders need to rethink how research positions are funded. Innovation is needed here but this review suggests that nothing is happening on this front.

Are the rationale for, and objectives of, the Systematic Review clearly stated?

Yes

Is the statistical analysis and its interpretation appropriate?

Not applicable

If this is a Living Systematic Review, is the ‘living’ method appropriate and is the search schedule clearly defined and justified? (‘Living Systematic Review’ or a variation of this term should be included in the title.)

Not applicable

Are sufficient details of the methods and analysis provided to allow replication by others?

Yes

Are the conclusions drawn adequately supported by the results presented in the review?

Yes

Reviewer Expertise:

Health behaviour, health communication, qualitative methods, systematic reviews

I confirm that I have read this submission and believe that I have an appropriate level of expertise to confirm that it is of an acceptable scientific standard.

# Response

**Published:** 2024-06-18

**Authors:** Amanda Blatch-Jones

**Affiliations:** Faculty of Medicine, University of Southampton, Southampton, Hampshire, UK

**Reviewer response:** In the Abstract, there are two stated aims, one under Background and one under Aims – these are not quite the same and I think it would be better to stick with one or the other (the first matches what is in the main text).

**Author response: **Thank you. We have replaced the aims with the following: The purpose of the scoping review was to explore ‘ *What does the evidence say about the ‘problem’ with ‘poor’ research culture, what are the benefits of ‘good’ research culture, and what does ‘good’ look like?’*

**Reviewer response: **Inclusion criteria or Types of sources – I think this needs a statement on the types of initiative that were studied e.g., ‘all initiatives aimed, partly or wholly, at improving research culture were included’. Or this may need to broader if reports were included about initiatives that did not specifically aim to improve research culture, but the impact on this was measured.

**Author response:** Thank you. We have included and revised your suggestion: All initiatives aimed, partly or wholly, at enhancing or assessing research culture were included.

**Reviewer response: **In the PRISMA flow diagram, the reasons for exclusion are only provided for the academic records – could they be added for the grey records too?

**Author response:** The flow diagram has been amended to include the exclusions and reasons.

**Reviewer response: **You mention that many of the articles were relevant to more than one of the four focus areas – could you add a little to the Methods to explain how you decided which area to categorise a report under in these cases? Or were they included in the synthesis for each category they related to (would be good to state this)?

**Author response:** We have added more detail about the four areas and how we allocated each article to an area or areas. “Both reviewers applied the articles under one of the four areas during extraction, based on the content and context of the article. Some articles were relevant to more than one category, which was reflected in the data extraction table. The reviewers independently checked the included articles to ensure that they were included appropriately across the four areas.”

**Reviewer response: **There is a typo in the fourth paragraph under ‘Research quality and accountability’ – ‘algin’?

**Author response: **Thank you. This has now been amended.

**Reviewer response: **Table 4 and Table 5 have the same title – I think Table 5 should be Key concepts and statements associated with research quality and accountability

**Author response: **Thank you for pointing this out. Table 5 title has been changed accordingly.

**Reviewer response: **It struck me that none of the concepts under ‘Security and career progression’ seemed to relate to job security, only career progression. This would be worth highlighting – ultimately to improve job security, research institutions and funders need to rethink how research positions are funded. Innovation is needed here but this review suggests that nothing is happening on this front.

**Author response: **Thank you. We have added the following sentence to the second paragraph under table 2. “To improve job security, research institutions and funding organizations need to readdress how research positions are funded, particularly in the early career stage.”

 Innovation was discussed in the some of the articles and these are reflected in the appropriate statements. We have tried to be inclusive to the areas that were most discussed, innovation for this area was more limited than the others (possibly due to challenges, barriers and competing interests around job security and progression). It is a valid point and thank you for bringing it up.

# Reviewer report: Reviewer response for version 1

**DOI:** 10.5256/f1000research.161809.r277506

**Published:** 2024-05-23

**Authors:** Steven De Peuter

**Affiliations:** 1 KU Leuven, Leuven, Flanders, Belgium

I have read this scoping review with interest. The authors have compiled and synthesized an impressive collection of works, both from academic sources and from popular sources. I was pleasantly surprised to see that they even included two podcasts.

 The authors not only review aspects of the research culture that are problematic. They also search the literature for evidence of what constitutes a good research culture and what can be done to promote it. That they limit their search to recently published material is not problematic: only recently has the shift been made from “codification and compliance” and from preventing the bad from happening towards promoting a healthy climate. It allows researchers and policy makers to get a better view on what is currently known about good research culture. The paper’s recommendations are clear and impressively complete, excellently summarizing the collected evidence, both in the text and in the clearly structured and comprehensive tables. The structure of the themes is also intuitive.

 I applaud the authors for their work and I want to emphasize that I consider this a relevant, solid, and quite exhaustive review. I have, however, a number of (minor) comments that I list below. Their purpose is solely to improve the relevance and reach of the paper, which I believe deserves a wide audience.

  

- Background, Discussion, Conclusions

The Background section is very much to the point. For readers who are familiar with the topic of research culture, it is sufficient to have the high-level overview of the main points that the paper currently provides. For interested readers who are less familiar with the topic the background section may be too high-level. The overview remains abstract and could benefit from more specific information and examples. For example, what is “the potential negative impact of a poor research culture / the consequences and challenges associated to an inadequate research culture”? What are some recommendations from the “international actions to address the underlying drivers of poor research culture”? (Which drivers are there?)

 Related to this, I think the Dicussion section would benefit from a brief recap of the main findings in the first paragraph(s), instead of just stating “From the evidence, it was clear that there were several initiatives to seek a cultural change across the research institutions/Higher Education Institutions.” The same applies to the conclusions. The authors correctly and rightfully emphasize the fact that “research institutions cannot act in isolation”, but in my opinion some of the findings deserve equal emphasis – and therefore being mentioned in the conclusions. In general, I think one of the conclusions could also be that there is *a lot* that can be done and that the evidence base for promoting a good research culture is substantial.

 When reading the paper, it felt a bit like I was reading a – very well written – internal report for policy makers who are knowledgeable of the potential problems with(in) research culture. I think it can be leveraged to also appeal to a broader audience.

  

- Methods

The *Types of sources* section confused me when I first read it, leading me to think that *only* empirical papers (“studies”) were included. Further on, it became evident that the authors included a wide variety of contributions. I suggest adding a first sentence to this section describing this, then continue by detailing the various types of studies and data that were included as it is currently documented.

  

- Results

The PRIMSA flow diagram (Figure 1) is incorrect. The total of the “Full-text articles excluded, with reasons (n = 513)” differs from the sum of the specific reasons (sum = 670). It appears that the latter is correct, because 924 papers screened – 670 excluded equals 254 included. Please note that there is still one paper that seems to have disappeared: Figure 1 and the main text mention 253 included papers, not 254.

 Unfortunately, in spite of the impressive amount of information that is confined in the Tables and despite my admiration for the work the authors must have put into presenting the evidence this way, there are inconsistencies between the number of references that are mentioned with the statements and the last column of the tables. You could consider this comment as an obsessive form of nitpicking from my part. I am aware of that. However, I believe that having a complete overview of the evidence associated with each statement will allow future readers to consult the relevant sources.

Table 2

 Theme *Promote fair and transparent process for career progression*

 Statement 2: number of references with statement = 39; last column = 35

 Statement 3: number of references with statement = 29; last column = 30

 Statement 6: number of references with statement = 20; last column = 21

 Theme *Reduce hyper competition and provide a culture of kindness*

 Statement 2: number of references with statement = 23; last column = 24

 Theme *Cultivate a culture of support that fosters a diverse set of skills and career pathways*

 Statement 1: number of references with statement = 33; last column = 34

 Statement 4: number of references with statement = 26; last column = 27

Table 3

 Theme *Embed and support an inclusive culture*

 Statement 1: number of references with statement = 44; last column = 43

 Statement 2: number of references with statement = 40; last column = 41

 Theme *Investing in people to reduce burden and improve wellbeing*

 Statement 1: number of references with statement = 39; last column = 42

 Statement 3: number of references with statement = 24; last column = 27

Table 4

 Theme *Everyone feeling valued and having equality of opportunities to contribute*

 Statement 2: number of references with statement = 25; last column = 24

Table 5

 Theme *Creation and facilitation*

 Statement 1: number of references with statement = 73; last column = 74

 Statement 2: number of references with statement = **54** (!!!); last column = 71

 Statement 3: number of references with statement = 45; last column = 46

 Statement 4: number of references with statement = 43; last column = 44

 Theme *Fostering transparency and visibility*

 Statement 2: number of references with statement = 49; last column = 51

 Statement 3: number of references with statement = 37; last column = 38

 Statement 6: number of references with statement = 12; last column = 13

  

- Study strengths and limitations

First paragraph: I would suggest to also include the percentage of articles from the USA, analogous to Canada and other international countries.

  

- General comments

Some of the sentences feel odd. For example, in *Wellbeing and equality of opportunity*, second paragraph, “The evidence suggests that these issues have an impact not only at the institutional level such as having a lack of diversity in organisational leaderships, but also for individuals, leading to a lack of role models and peer mentors skills shortages in particular disciplines, sectors and roles, and drives off talent.” is a run-on and feels like a collation of several sentences. Please check.

 Some of the Statements in the tables are also long and may better be divided into several shorter sentences. Some examples:

 Table 2, Statement 3 under theme *Reduce hyper competition and provide a culture of kindness*

 “Consider ways to demonstrate support for other researchers to secure funding as part of progression (and review current reward systems), ensuring education, teaching and research are equally prioritised”: who is referred to by “other”?

 Table 5, under theme *Fostering transparency and visibility* Statements 2 and 7 feel odd. It seems like parts of the statements are missing. Please reformulate.

  

- Typo’s

**Abbreviations list**

 COPE is the “Committee on Publication Ethics”, not “of” – the same typo is in Table 5 (theme “Fostering transparency and visibility” number 3 “Gain greater understanding…”)

 ENRI: do the authors mean European Network of Research Integrity *Offices (ENRIO* - https://www.enrio.eu/ *)*, or maybe *ENERI*, the “European Network for Research Ethics and Integrity” ( https://eneri.eu)? If corrected, please also correct the mention of ENRI in Table 5 (same place as COPE) and in the last paragraph before the Discussion section.

 KPI à capitalize “Performance”

**Background**

 3d paragraph: The consequences of poor research culture does (change into “do”) not only impact researchers, it also effects (change into “affects”) research support staff…

**Results**

*Security and career progression* 3d paragraph: “ *The review suggests that the problem is reinforced by a culture where researchers are incentivized to produce many funding applications and academic publications where high rejection rates*” this sentence seems to be missing a verb.

*Research quality and accountability – open and trustworthy research *4th paragraph: change “algin” into “align”

**Tables**

 In Table 2, under theme *Cultivate a culture of support that fosters a diverse set of skills and career pathway *the references of Statement 8 are not included as links (they are also between brackets). This may be a formatting problem beyond the authors’ control, though.

 In Table 3, under theme *Embed and support an inclusive culture *Statement 6: …including shift (change into “shifting”) institutional practice…

 In Table 3, under theme *Making use of and learning from existing tools and initiatives to support cultural change* there is no space before the opening bracket in Statement 2

 In Table 5, under theme *Incentives and innovation* Statement 1 mentions (at the end) “open assess movement”. Do the authors maybe mean “open access movement”?

 In Table 5, under theme *Fostering transparency and visibility* Statement 5 please change “CREDiT” into “CRediT”

**Other**

 Seeing “life-work balance” mentioned in Table 3 made me smile. However, in the section *Wellbeing and equality of opportunity* and in the Discussion section the authors shift to work-life balance, also using “work/life balance” in the discussion. Please be consistent.

Are the rationale for, and objectives of, the Systematic Review clearly stated?

Yes

Is the statistical analysis and its interpretation appropriate?

Not applicable

If this is a Living Systematic Review, is the ‘living’ method appropriate and is the search schedule clearly defined and justified? (‘Living Systematic Review’ or a variation of this term should be included in the title.)

Not applicable

Are sufficient details of the methods and analysis provided to allow replication by others?

Yes

Are the conclusions drawn adequately supported by the results presented in the review?

Yes

Reviewer Expertise:

Research integrity, research culture, behavior change, health psychology, change management

I confirm that I have read this submission and believe that I have an appropriate level of expertise to confirm that it is of an acceptable scientific standard, however I have significant reservations, as outlined above.

# Response

**Published:** 2024-06-18

**Authors:** Amanda Blatch-Jones

**Affiliations:** Faculty of Medicine, University of Southampton, Southampton, Hampshire, UK

Background: The overview remains abstract and could benefit from more specific information and examples. For example, what is “the potential negative impact of a poor research culture / the consequences and challenges associated to an inadequate research culture”? What are some recommendations from the “international actions to address the underlying drivers of poor research culture”? (Which drivers are there?)

**We have amended the first paragraph to provide more content and greater clarity for the reader.**

 Related to this, I think the Discussion section would benefit from a brief recap of the main findings in the first paragraph(s), instead of just stating “From the evidence, it was clear that there were several initiatives to seek a cultural change across the research institutions/Higher Education Institutions.”

**We have added to the first part of the discussion emphasising some of the key points as suggested.**

 The same applies to the conclusions. The authors correctly and rightfully emphasize the fact that “research institutions cannot act in isolation”, but in my opinion some of the findings deserve equal emphasis – and therefore being mentioned in the conclusions. In general, I think one of the conclusions could also be that there is *a lot* that can be done and that the evidence base for promoting a good research culture is substantial.

**Thank you for your comments and we have included your suggested conclusion comment into the manuscript. We have also amended the sentence “research institutions cannot act in isolation.”**

 When reading the paper, it felt a bit like I was reading a – very well written – internal report for policy makers who are knowledgeable of the potential problems with(in) research culture. I think it can be leveraged to also appeal to a broader audience.

**Thank you for your feedback. We have made notable changes to the manuscript which we hope provides greater coverage for a broader audience.**

 Methods: The *Types of sources* section confused me when I first read it, leading me to think that *only* empirical papers (“studies”) were included. Further on, it became evident that the authors included a wide variety of contributions. I suggest adding a first sentence to this section describing this, then continue by detailing the various types of studies and data that were included as it is currently documented.

**Thank you for your suggestion. We have included an opening sentence for this sub-heading. “Several types of contributions were used for the scoping review, which included articles, reports, blogs and web-based articles from both empirical studies and grey literature.”**

 Results: The PRIMSA flow diagram (Figure 1) is incorrect. The total of the “Full-text articles excluded, with reasons (n = 513)” differs from the sum of the specific reasons (sum = 670). It appears that the latter is correct, because 924 papers screened – 670 excluded equals 254 included. Please note that there is still one paper that seems to have disappeared: Figure 1 and the main text mention 253 included papers, not 254.

 Unfortunately, in spite of the impressive amount of information that is confined in the Tables and despite my admiration for the work the authors must have put into presenting the evidence this way, there are inconsistencies between the number of references that are mentioned with the statements and the last column of the tables. You could consider this comment as an obsessive form of nitpicking from my part. I am aware of that. However, I believe that having a complete overview of the evidence associated with each statement will allow future readers to consult the relevant sources.

**Thank you for pointing this out on the PRISMA flow diagram. Having gone back to the reference manager the error has been corrected. The number of exclusions were 671 and the missing article was not recorded. This has now been amended.**

**Thank you for your thoughtful comment and paying particular attention to the mismatch between the number of references cited for each statement and the count of these references in the tables. We really appreciate your attention to detail and have made the relevant changes and have double checked these for consistency.**

Table 2

 Theme *Promote fair and transparent process for career progression*

 Statement 2: number of references with statement = 39; last column = 35

 Statement 3: number of references with statement = 29; last column = 30

 Statement 6: number of references with statement = 20; last column = 21

 Theme *Reduce hyper competition and provide a culture of kindness*

 Statement 2: number of references with statement = 23; last column = 24

 Theme *Cultivate a culture of support that fosters a diverse set of skills and career pathways*

 Statement 1: number of references with statement = 33; last column = 34

 Statement 4: number of references with statement = 26; last column = 27

**Thank you. We have adjusted the frequencies and have doubled checked all the other statements for clarity.**

Table 3

 Theme *Embed and support an inclusive culture*

 Statement 1: number of references with statement = 44; last column = 43

 Statement 2: number of references with statement = 40; last column = 41

 Theme *Investing in people to reduce burden and improve wellbeing*

 Statement 1: number of references with statement = 39; last column = 42

 Statement 3: number of references with statement = 24; last column = 27

**Thank you. We have adjusted the frequencies and have doubled checked all the other statements for clarity.**

Table 4

 Theme *Everyone feeling valued and having equality of opportunities to contribute*

 Statement 2: number of references with statement = 25; last column = 24

**Thank you. We have adjusted the frequencies and have doubled checked all the other statements for clarity.**

Table 5

 Theme *Creation and facilitation*

 Statement 1: number of references with statement = 73; last column = 74

 Statement 2: number of references with statement = **54** (!!!); last column = 71

 Statement 3: number of references with statement = 45; last column = 46

 Statement 4: number of references with statement = 43; last column = 44

 Theme *Fostering transparency and visibility*

 Statement 2: number of references with statement = 49; last column = 51

 Statement 3: number of references with statement = 37; last column = 38

 Statement 6: number of references with statement = 12; last column = 13

**Thank you. We have adjusted the frequencies and have doubled checked all the other statements for clarity.**

 Study strengths and limitations: First paragraph: I would suggest to also include the percentage of articles from the USA, analogous to Canada and other international countries.

**These figures and sentence has been adjusted to reflect the accuracy of the percentages in table 1.**

 General comments: Some of the sentences feel odd. For example, in *Wellbeing and equality of opportunity*, second paragraph, “The evidence suggests that these issues have an impact not only at the institutional level such as having a lack of diversity in organisational leaderships, but also for individuals, leading to a lack of role models and peer mentors skills shortages in particular disciplines, sectors and roles, and drives off talent.” is a run-on and feels like a collation of several sentences. Please check. (1)

 Some of the Statements in the tables are also long and may better be divided into several shorter sentences. Some examples:

 Table 2, Statement 3 under theme *Reduce hyper competition and provide a culture of kindness*

 “Consider ways to demonstrate support for other researchers to secure funding as part of progression (and review current reward systems), ensuring education, teaching and research are equally prioritised”: who is referred to by “other”? (2)

 Table 5, under theme *Fostering transparency and visibility* Statements 2 and 7 feel odd. It seems like parts of the statements are missing. Please reformulate. (3 and 4)

**Thank you for your general comments on some of the sentences in the manuscript. We have amended these, which hopefully makes more sense for the reader.**

  

1. **“The evidence suggests that these wellbeing issues can have an impact at an institutional level, resulting in a lack of diversity across leadership roles, a shortage of role models and peer mentors, and driving off talent due to staff leaving academia.”**
2. **‘other’ has been taken out as that was a typo error. Thank you for drawing us to this statement.**

**Due to the structure of the statements and how they emerged as part of the synthesis, it would be challenging to go back and re-evaluate these statements. We understand that some of them are longer than others, as we attempted to capture sufficient information to enable the reader to understand the content and context.**

**Statement 2 has been amended “Actively encouraging researchers to make their research more accessible and open through sharing protocols and data openly and transparently, could foster greater knowledge exchange opportunities.”**

**Statement 7 has also been amended “Become a signatory of initiatives such as DORA and seek to engage with local and international networks such as the Reproducibility Network.”**

 Typo’s:

**Abbreviations list**

 COPE is the “Committee on Publication Ethics”, not “of” – the same typo is in Table 5 (theme “Fostering transparency and visibility” number 3 “Gain greater understanding…”)

 ENRI: do the authors mean European Network of Research Integrity *Offices (ENRIO* - https://www.enrio.eu/ *)*, or maybe *ENERI*, the “European Network for Research Ethics and Integrity” ( https://eneri.eu)? If corrected, please also correct the mention of ENRI in Table 5 (same place as COPE) and in the last paragraph before the Discussion section.

 KPI à capitalize “Performance”

**Background**

 3d paragraph: The consequences of poor research culture does (change into “do”) not only impact researchers, it also effects (change into “affects”) research support staff…

**Results**

*Security and career progression* 3d paragraph: “ *The review suggests that the problem is reinforced by a culture where researchers are incentivized to produce many funding applications and academic publications where high rejection rates*” this sentence seems to be missing a verb.

*Research quality and accountability – open and trustworthy research *4th paragraph: change “algin” into “align”

**Tables**

 In Table 2, under theme *Cultivate a culture of support that fosters a diverse set of skills and career pathway *the references of Statement 8 are not included as links (they are also between brackets). This may be a formatting problem beyond the authors’ control, though.

 In Table 3, under theme *Embed and support an inclusive culture *Statement 6: …including shift (change into “shifting”) institutional practice…

 In Table 3, under theme *Making use of and learning from existing tools and initiatives to support cultural change* there is no space before the opening bracket in Statement 2

 In Table 5, under theme *Incentives and innovation* Statement 1 mentions (at the end) “open assess movement”. Do the authors maybe mean “open access movement”?

 In Table 5, under theme *Fostering transparency and visibility* Statement 5 please change “CREDiT” into “CRediT”

**Other**

 Seeing “life-work balance” mentioned in Table 3 made me smile. However, in the section *Wellbeing and equality of opportunity* and in the Discussion section the authors shift to work-life balance, also using “work/life balance” in the discussion. Please be consistent.

**Thank you for your additional suggestions on the typo’s in the manuscript. We have addressed all your comments accordingly.**

**For the references, on statement 8, it is a formatting issue outside of our control (a request to F1000 has been made in the revised manuscript)**
